# Integrative omics analysis in *Pandanus odorifer* (Forssk.) Kuntze reveals the role of Asparagine synthetase in salinity tolerance

**DOI:** 10.1038/s41598-018-37039-y

**Published:** 2019-01-30

**Authors:** Deo Rashmi, Vitthal T. Barvkar, Altafhusain Nadaf, Swapnil Mundhe, Narendra Y. Kadoo

**Affiliations:** 1 Department of Botany, Savitribai Phule Pune University, Pune, 411007 India; 2 Biochemical Sciences Division, CSIR-National Chemical Laboratory, Pune, 411008 India

## Abstract

*Pandanus odorifer* (Forssk) Kuntze grows naturally along the coastal regions and withstands salt-sprays as well as strong winds. A combination of omics approaches and enzyme activity studies was employed to comprehend the mechanistic basis of high salinity tolerance in *P*. *odorifer*. The young seedlings of *P*. *odorifer* were exposed to 1 M salt stress for up to three weeks and analyzed using RNAsequencing (RNAseq) and LC-MS. Integrative omics analysis revealed high expression of the Asparagine synthetase (AS) (EC 6.3.5.4) (8.95 fold) and remarkable levels of Asparagine (Asn) (28.5 fold). This indicated that salt stress promoted Asn accumulation in *P*. *odorifer*. To understand this further, the Asn biosynthesis pathway was traced out in *P*. *odorifer*. It was noticed that seven genes involved in Asn bisynthetic pathway namely glutamine synthetase (GS) (EC 6.3.1.2) glutamate synthase (GOGAT) (EC 1.4.1.14), aspartate kinase (EC 2.7.2.4), pyruvate kinase (EC 2.7.1.40), aspartate aminotransferase (AspAT) (EC 2.6.1.1), phosphoenolpyruvate carboxylase (PEPC) (EC 4.1.1.31) and AS were up-regulated under salt stress. *AS* transcripts were most abundant thereby showed its highest activity and thus were generating maximal Asn under salt stress. Also, an up-regulated Na^+^/H^+^ antiporter (NHX1) facilitated compartmentalization of Na^+^ into vacuoles, suggesting *P*. *odorifer* as salt accumulator species.

## Introduction

Plants are sessile organisms and consistently subjected to a vast range of environmental stresses. In order to survive and sustain these stresses various physiological and molecular rearrangements take place in the cellular machinery of a plant system. Salinity is one of the major causes of huge loss of crop productivity arising mainly due to natural or anthropogenic activities^1^. It is expected that by the year 2050 nearly 50% of the cultivable land would be salinized due to climate change^2^. Nonetheless, the plants adapted to saline environments like halophytes can successfully defend the stressful conditions and maintain integrity of their cellular systems through multiple mechanisms^3,4^. Although several salt tolerance genes have been identified and characterized from them, the exact mode of survival of halophytes on saline soils is largely unclear^5^. This has been primarily due to the non-availability of genomic resources of halophytes. RNAsequencing is one of the most powerful and frequently used tools for the comparative transcriptomic analysis, especially in species lacking reference genomes^6^. Likewise, extensive investigation of variation in metabolite levels can be performed via total metabolic profiling followed by multivariate statistical analyses, which are collectively called metabolomics^7^. For determining the gene to metabolite correlation, combined non-targeted transcriptome and metabolome analyses are most useful and feasible tools. Since the ultimate downstream product of gene transcription is metabolome, the magnifications in the metabolic changes are relative to those in the transcriptome^8^. The integration of these allows identification of functions of known/ unknown genes in the cells of the concerned biological system taken for study.

Under salt stress, tolerant plants tend to accumulate ions, adjust osmotic parameters, maintain water potential and synthesize as well as accumulate osmoprotectant molecules to survive and sustain the osmotic shock^9^. Despite the decrease in soil-water potential under salinity, osmolytes allow water absorption by lowering the cell osmotic potential. As osmolytes can be accumulated at any stage of plant development, they are beneficial in terms of scavenging the reactive oxygen species and also protecting sub-cellular structures^10^. The diversity of osmolytes is very broad which includes amino acids (proline, alanine, Asn, GABA, pipecolic acid etc.), sugars (sucrose, trehalose, etc.) and quaternary ammonium compounds to name a few^10^.

The amino acid Asparagine (Asn) is accumulated in several plant species under abiotic stress conditions such as salt & drought stress and mineral deficiency etc.^11,12^. The osmolytic properties of Asn have been justified with evidence^13,14^. Asn is synthesized by glutamine-dependent asparagine synthetase (AS: EC 6.3.5.4) that catalyzes the transfer of an amide group from glutamine (Gln) to aspartate (Asp) in ATP-dependent manner. Frequent accumulation of Asn in plants has been repeatedly reported under salt stress^15^ while other amides are barely found accumulating^11^.

*Pandanus odorifer* (Forssk.) Kuntze, commonly known as screw-pine, is a monocot species that belongs to the family of Pandanaceae and grows naturally alongside the coastal regions of Asia, South-east Asia and Polynesia. The plants are dioecious and highly fertile bearing flowers and fruits. The consistent saline sprays barely have any effect on their productivity^16^. With the support of aerial roots or prop roots their leaves often reach an elevation of 20 meters. Owing to the density and complex aerial root structures, they are found effective in protection against tsunami damages^16^. The essential oil obtained from male inflorescence bracts is aromatic. It is traditionally used in the treatment of headaches, ear-aches, rheumatic pains and several skin diseases apart from being used as a natural flavouring agent. Various health benefits of the species include treatment of fever, leprosy, smallpox and wounds. Along with its function as a laxative for children it also eases chest pains as well as reduces stomach spasms and strengthens the gum^16^.

Previously, we discussed the role of GABA as an osmolyte in *P*. *odorifer*^17^. However, it is observed that when the *P*. *odorifer* seedlings are treated with high concentration of salt, metabolic shift from GABA to Asn could be recorded through integrative omics approaches. The analysis reveals up-regulation of *AS* (encoding asparagine synthetase), increased activities of enzymes related to Asn synthesis, as well as increased accumulation of the metabolite Asn in response to salt-stress establishing a key role of Asn as an osmolyte in *P*. *odorifer* to manage the salt stress. AS gene from *P*. *odorifer* could be transferred to other crops to enable their cultivation on saline soils.

## Results and Discussion

Recent advances have made it possible to integrate various technologies in order to elucidate the roles of genes and metabolites. It can be done with the help of extensive analysis of gene expression through transcriptomics and changes in accumulation of metabolites through metabolomics. In this study, we treated three month old seedlings of *P*. *odorifer* with 1 M salt concentration for three weeks and analyzed the changes in gene expression and metabolite accumulation through transcriptomics and metabolomics approaches. At the end of three weeks, after initiating the salt treatment, *P*. *odorifer* seedlings started showing wilting symptoms above 1 M salt concentrations. Therefore, 1 M NaCl concentration for 3 weeks was considered as tolerable concentration for *P*. *odorifer* seedlings. The transcriptomics and metabolomics analyses were performed with respect to various parameters at this concentration results of which are discussed below.

### Comparative de novo transcriptomics analysis in control and salt treated *P*. *odorifer* seedlings

Comparative *de novo* assembly and quantitative assessment of IIIumina RNAseq data of control and 1 M salt treated seedlings of *P*. *odorifer* was performed. A total of 56.59 Gb raw data comprising 56,589,112 raw reads were obtained for both the libraries and were considered together. After removing low-quality regions and sequences, adapters and all possible contamination, a total of 49,340,809 clean reads with Q30 > 95% (error probability <= 0.001) and GC percentage between 43.59% − 45.95% were obtained (Table 1). Each stage was represented by more than 23 million high-quality reads, ranging from 23,779,040 to 25,561,769 confirming that the sequence output and quality statistics were adequate for use in further analysis. After assembling, a total of 298,438 transcript sequences were produced through Trinity v2.2^6^, that has an average transcript size of 951 bp and N50 of 1821 bp (Supplementary Table S1, Sheet1). Further, the CD-HIT tool (v4.6.1)^18^ was employed at 90% identity to obtain 244,033 non-redundant transcripts and about 89.32% of the paired end reads got back aligned, indicating that significant proportion of reads were represented in the Trinity assembly. The quality of these assemblies and transcript length distribution and sequence similarity distributions are presented in Supplementary Figs. S1 and S2 respectively. For 20,475 unigenes, the sequence length was in the range of 1.0–1.5 Kb, while for 13,029 unigenes, it was in the range of 1.5–2.0 Kb. It shows that nearly full length transcripts were obtained for most of the genes. GC content (45.95%) of salt-treated tissues of *P*. *odorifer* may be attributed to the ability to adapt in extreme environment, besides reflecting high quality sequencing run (Supplementary Fig. S3)^19^.

**Table 1:** Primary assembly statistics in control and 1 M NaCl treated *P*. *odorifer* libraries

Parameters	Control	1 M salt treated
Total number of reads	28,990,762	27,598,350
Total number of HQ reads	25,561,769	23,779,040
Percentage for HQ reads	88.17	86.16
Mean read length (bp)	100	100
GC%	43.59	45.95

### Functional annotation and classification of genes expressed upon salt treatment in *P*. *odorifer*

The analysis revealed that about 73,827 (54.20%) transcripts significantly matched the known genes in NCBI nt database (https://www.ncbi.nlm.nih.gov/nucleotide/). The E-value distribution (Supplementary Fig. S4) of the top hits in the NCBI nr database (https://www.ncbi.nlm.nih.gov/protein/) showed that around 59% of the transcripts found using BLASTX have confidence level of at least 1e^−10^. About 74% of the assembled transcripts had similarity of more than 60% at protein level with the existing proteins in NCBI database. Approximately 96% of the annotated transcripts could be assigned with the best score to the sequences from the top six species *viz*., *Elaeis guineensis*, *Phoenix dactylifera*, *Musa acuminata*, *Anthurium amnicola*, *Ananas comosus* and *Nelumbo nucifera* (Supplementary Fig. S5). The annotated transcripts mostly matched the transcripts from species that grow in moderately saline habitats. This suggests that the EST dataset could be used as a valuable transcriptome resource for further gene discovery and functional analysis.

### Functional categorization of genes expressed in *P*. *odorifer* under 1 M salt treatment

Gene ontology (GO) is widely used to provide global overview of functional analysis and prediction of biological significance of transcriptomic datasets. A total of 13,133 transcripts were assigned at least one GO term to describe the biological processes, molecular functions and cellular components (Supplementary Fig. S6a, b and c).

### GO analysis of differentially expressed genes (DEGs)

The dominant terms in biological processes group of GO for salt treated *P*. *odorifer* were biological process, cellular process, single-organism process and response to stimulus (Supplementary Fig. S6a). Others were in response to stress, abiotic stimulus, organic substances, salt stress etc. In the cellular components, the dominant terms were cytosol, plasma membrane, membrane, chloroplast and mitochondrion (Supplementary Fig. S6b) and remaining included cytoplasmic part, cell wall, cytoplasm, vacuole, Golgi apparatus etc. In molecular functions, the dominant terms were catalytic activity, protein binding, transferase activity, molecular function, and ATP binding (Supplementary Fig. S6c). Some others were copper ion binding, cobalt ion binding, nucleic acid binding, ubiquitin protein transferase activity, etc.

The GO terms that were identified during salt stress treatment in *P*. *odorifer* are the ones which have been mostly reported upon analyses of DEGs under salt stress conditions in different plants particularly halophytes and xerophytes^4,20^.

### KEGG pathway annotation of DEGs in 1M salt treated *P*. *odorifer*

For the KEGG pathway annotation of DEGs, only those with FPKM (fragments per kilobase per million) greater than 10 were considered. Total number of 1891 sequences was obtained (Supplementary Table S1, Sheet2), out of which 384 non-redundant transcripts were assigned to 41 pathways (Supplementary Table S1, Sheet3). Signal transduction represented the largest group (10.4%) followed by carbohydrate metabolism (7.86%), amino acid metabolism (7.29%), carbon metabolism (5.14%) and lipid metabolism (4.86%).

### Transcription factors (TFs) expressed under salt stress in *P*. *odorifer*

Transcription factors (TFs) are the major regulatory elements. They play significant role in gene expression, plant secondary metabolism and response to environmental stresses by binding to specific cis-regulatory elements of promoter regions^21^ and modulating gene expression. Homology search (E value < e^−10^ and percent identity >90) of assembled transcriptome was carried out to understand the diversity of TFs up-regulated in salt treated *P*. *odorifer*. In present study, a total of 6,334 (~5%) transcripts were assigned to 15 different TF families. Among these, NAC (1, 263), ERF (985), MYB related (583), DREB (582), IAA16 (448) and WRKY (375) were the most abundant (Supplementary Table S1, Sheet2). Moreover, TF families, up-regulated under salt stress in *P*. *odorifer*, were reported to be involved in regulation at molecular levels under abiotic and biotic stress responses in several plant species^22^.

### Expression profiles of transcripts involved in conferring salt tolerance in *P*. *odorifer*

**Figure 1:**
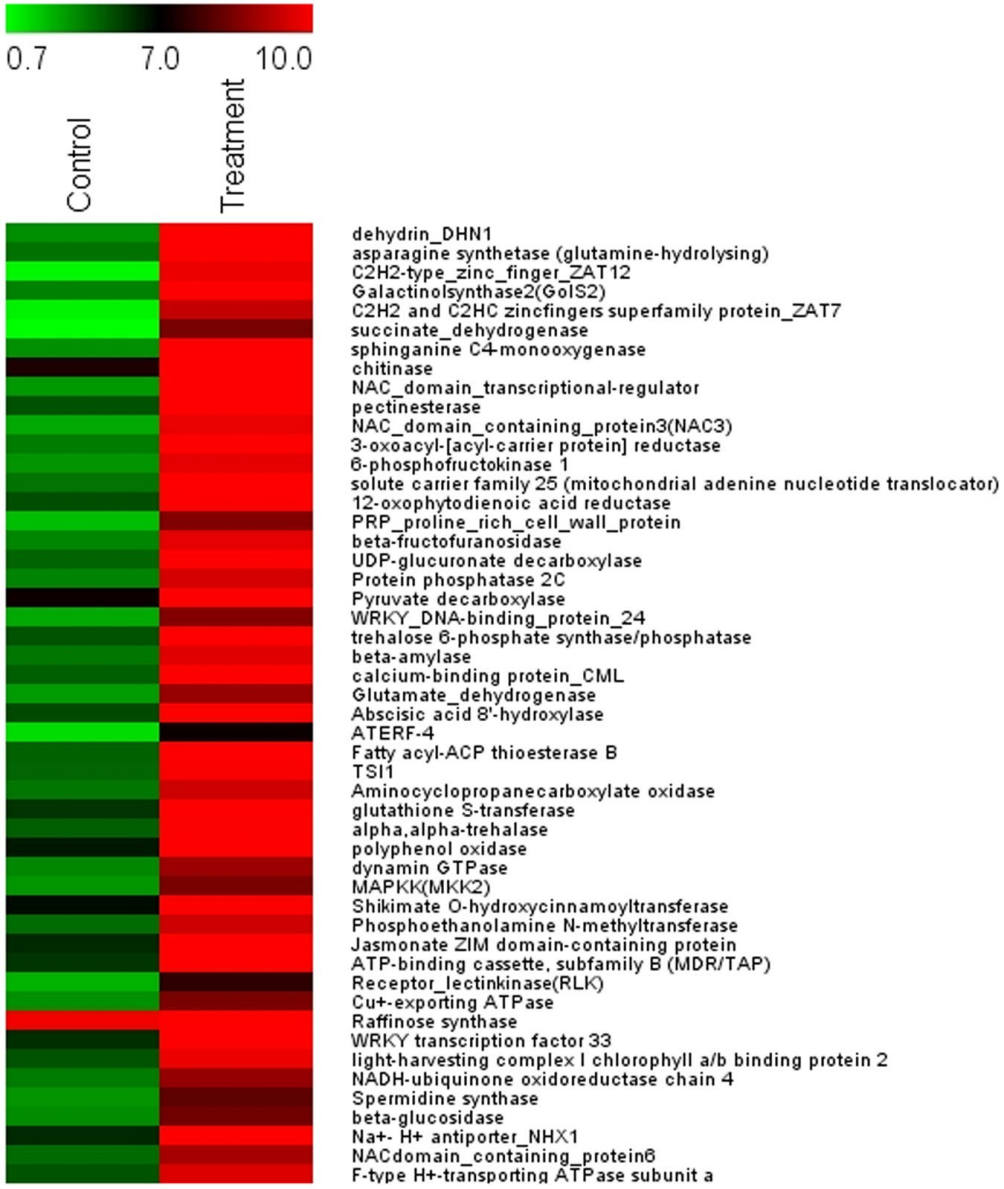
Heat map representing expression dynamics of top 50 up-regulated transcripts (expressed as log2 fold change ratio) in 1 M salt treated *P*. *odorifer*. Transcript names are given on the right side. Color scale representing normalized expression values is shown at the top.

**Figure 2:**
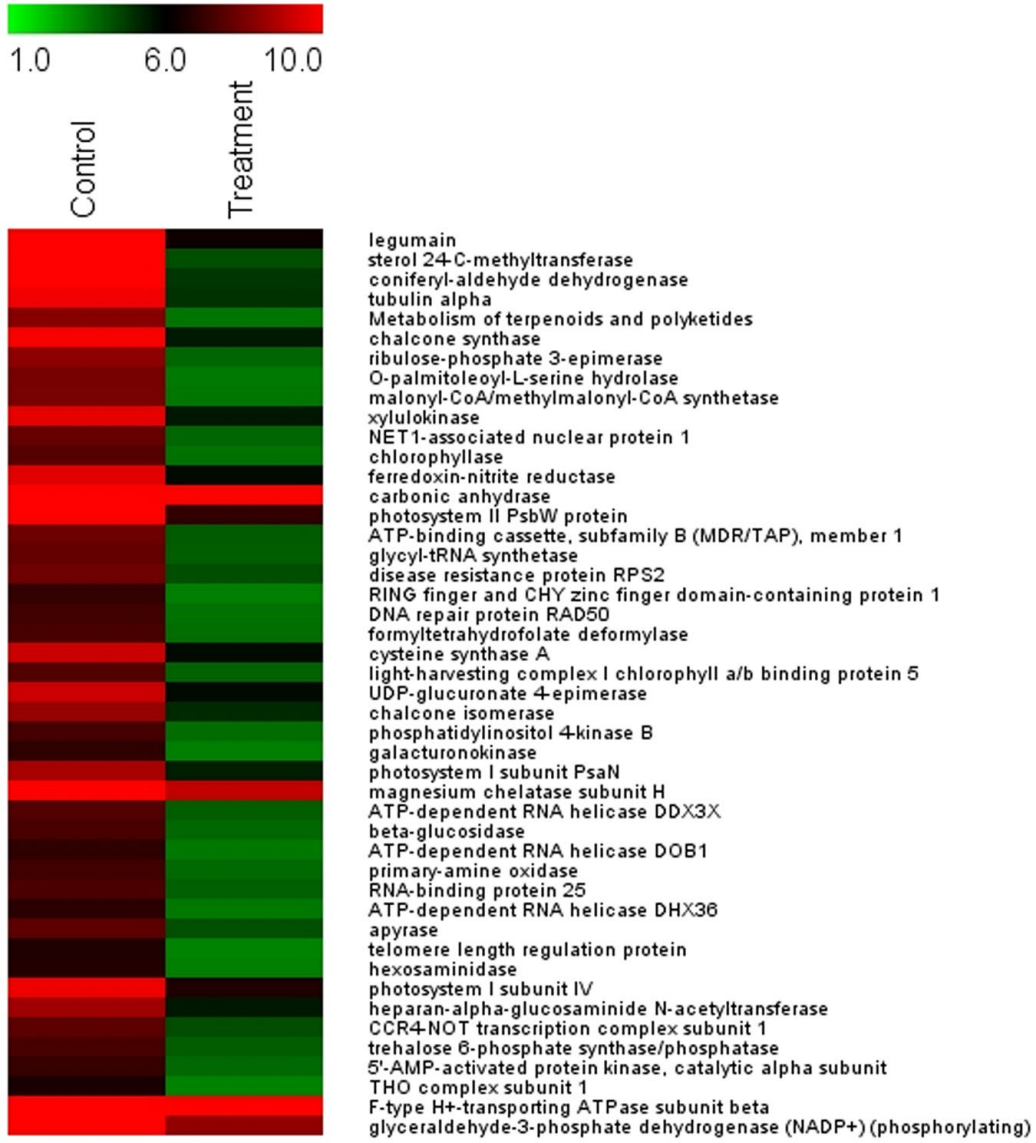
Heat map representing expression profiles of 50 most down-regulated transcripts (expressed as log2 fold change ratio) in 1 M salt treated *P*. *odorifer*. Transcript names are given on the right side. Color scale representing normalized expression values is shown at the top.

Top fifty differentially expressed transcripts (expressed as log2 fold change ratio) are displayed in the heat maps (Figs. 1 and 2). The up-regulated genes, in response to salt treatment, included the ones that played major biochemical regulatory roles: the genes encoding osmoprotectants; TFs, proteins that are involved in ubiquitin-mediated modifications of proteins and their degradation. Also Enzymes and proteins facilitating the synthesis of hormones, molecular chaperons facilitating protein folding and stabilization of the secondary structure of proteins and proteins involved in post-transcriptional modifications of mRNAs, etc. (Fig. 1).

Likewise, the heat map in Fig. 2 reveals major down-regulated genes under salt stress in *P*. *odorifer*. The most down-regulated genes included legumain (−1.05 fold), sterol methyl transferase (−1.20 fold), apyrase (−0.81 fold) and RNA helicase (−0.877). Legumain belongs to the family of Asn-specific cysteine proteinases, which functions as propolypeptide-processing or protein-degrading enzyme. They are also important in triggering programmed cell death in plants due to caspase-like activity and similarity of active sites^23^. Therefore, their down-regulation in salt treated *P*. *odorifer* indicates plant’s well-being. The results indicate that Asparagine synthetase (*AS*), encoding the osmolyte Asn, ranked the second highest among the up-regulated genes. Hence we narrowed down our further analysis to *AS*.

### Putative salt tolerance genes

Some of the candidate genes that were highly up-regulated under salt stress in *P*. *odorifer*, as discovered via heat map analysis, were DHN (8.99 fold), AS (8.95 fold), ZFPs (8.90 fold), galactinol sythetase (8.67 fold), succinate dehydrogenase (7.75 fold), chitinase (7.27 fold) and NAC domain TFs (7.08 fold). The genes responsible for Na^+^ compartmentalization like Na^+^/H^+^ antiporter (NHX1) (4.7 fold) and V type H^+^ transporting ATPases (1.4 fold) were also up-regulated under salt stress in *P*. *odorifer*.

Most up-regulated genes in *P*. *odorifer* under salt treatment are dehydrin, which belongs to group II late embryogenesis abundant protein (LEA) and AS; both are known to be osmoprotectants. DHNs are the multi-family proteins produced by plants in response to multiple environmental stresses^24^. AS generate the metabolite asparagine (Asn) from aspartate. It is also a known osmolyte in plant systems^10^. This result in itself highlights and supports the dominant role of osmoprotectants played under salt stress in *P*. *odorifer*. Dehydrins and Osmotins were highly expressed under salt treatment^3^ in halophyte *Suaeda fruticosa* too. In *P*. *odorifer*, other up-regulated genes like ZFPs and those belonging to a super family of proteins, were often found regulating responses to various biotic and abiotic stresses^25^. Galactinol synthase, which is a key regulator of the biosynthesis of Raffinose family oligosaccharides, accumulates under abiotic stresses^26^. Plant chitinases have also been found playing a versatile role from providing higher resistance to pathogens to physiological processes like, growth and developmental regulation, programmed cell death, symbiosis and environmental stress tolerance^27^. Likewise, it is known that NAC domain TFs play significant role in regulating plant growth and development processes including abiotic stress responses^28^.

Besides this, several transcripts specifically induced by salt treatment and expressed in enrichment pathways, were identified such as the integral component of membrane, adenosine triphosphate (ATP) binding, protein serine threonine kinase activity, nucleus, carbohydrate metabolism, transcription, and TF activity etc. (Supplementary Fig. S7).

### Significant expression modulation in asparagine metabolic pathway genes

**Figure 3:**
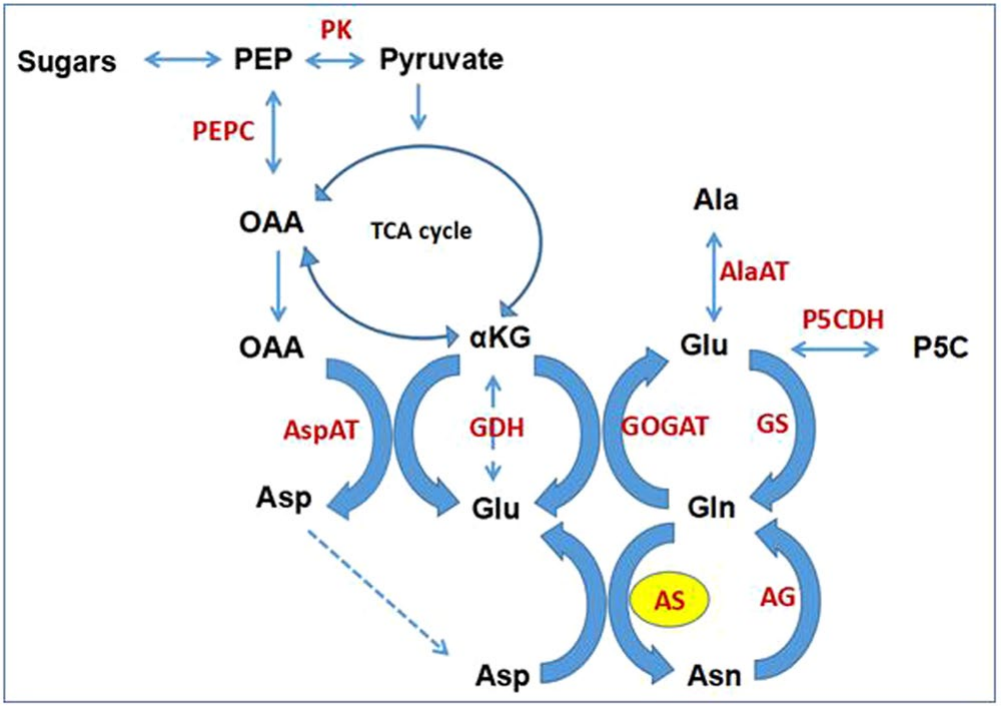
Proposed Asparagine metabolic pathway in *P*. *odorifer* under salt stress (Legend:- PEP: Phosphoenol pyruvate, OAA: Oxaloacetate, αKG: α-keto glutarate, Asp: Aspartate, Glu: Glutamate, Gln: Glutamine, Asn: Asparagine, Ala: Alanine, P5C: 1-Pyrroline-5-carboxylate, PEPC: Phosphoenolpyruvate carboxylase, PK: Pyruvate kinase, AspAT: Aspartate amino transferase, GDH: Glutamate dehydrogenase, GOGAT: Glutamate synthase, GS: Glutamine synthetase, AlaAT: Alanine amino transferase, P5CDH: P5C dehydrogenase, AG: Asparaginase, AS: Asparagine synthetase).

AS was nearly 8.95 folds up-regulated under 1 M salt treatment (Fig. 1). The biosynthesis of Asn in higher plants is primarily mediated via Gln dependent pathway (Fig. 3). Asn is synthesized via amide transfer from Gln to Asp with the help of AS. Asp is derived from transamination of oxaloacetate (OAA) and glutamate (Glu) through the action of aspartate aminotransferase (AspAT; EC 2.6.1.1). Production of OAA is versatile; it can be synthesized from pyruvate by the anaplerotic reaction to replenish the TCA cycle intermediates, as well as by the activity of phosphoenolpyruvate carboxylase (PEPC; EC 4.1.1.31) from phosphoenolpyruvate. NH_4_^+^ is mainly assimilated into Gln and Glu via joint activities of glutamine synthetase (GS; EC 6.3.1.2) and glutamate synthase (GOGAT; EC 1.4.1.14), known as the GS/GOGAT cycle. Glutamate dehydrogenase (GDH; EC 1.4.1.2) also assimilates NH_4_^+^ into Glu from α keto-glutarate (αKG). The resulting Glu can be incorporated into Asp and Asn via AS^29^. The enzyme asparaginase (AG; EC 3.5.1.1) carries out a hydrolysis of Asn, forming Asp and NH_3_, which re-assimilates through GS-GOGAT cycle^30^.

In this study, we identified a total of nine genes that were involved in Asn bisynthetic pathway namely alanine synthesizing transaminase, GS, GOGAT, aspartate kinase, pyruvate kinase, AspAT, PEPC, AS and AG. These genes were up- or down-regulated under salt stress (Table 2). *AS* transcripts were most abundant (8.95 fold) thereby showing highest activity therefore generating maximal Asn. AspAT transcripts were 4.15 fold up-regulated and facilitated synthesis of Asp from OAA. An up-regulated PEPC (3.44 fold) aided formation of OAA from PEP. The high levels of NH_4_^+^ assimilating enzymes GS (3.97 fold) and GOGAT (2.57 fold) also maintained the flux towards formation of Asn. Along with them GDH (3.09 fold) was found incorporating NH_4_^+^ towards Glu from TCA intermediate (αKG). Lastly, the rate of catabolism of Asn was low as AG was down-regulated at this point.

**Table 2:** FPKM values of genes involved in Asn biosynthesis under 1 M salt treatment in *P*. *odorifer*

Gene	Control	1 M Salt treatment
Asparagine synthetase	18.00	8891.00
Alanine synthesizing transaminase	146.46	2797.39
Glutamine synthetase	29.70	413.21
Glutamate synthase	6727.00	3572.00
Aspartate kinase	62.38	575.53
Pyruvate kinase	48.08	396.17
Aspartate aminotransferase	56.70	886.42
Phosphoenolpyruvate carboxylase	40.35	378.97
Asparaginase	15.00	11.00

### Validation of transcriptomics results through qRT-PCR

**Figure 4:**
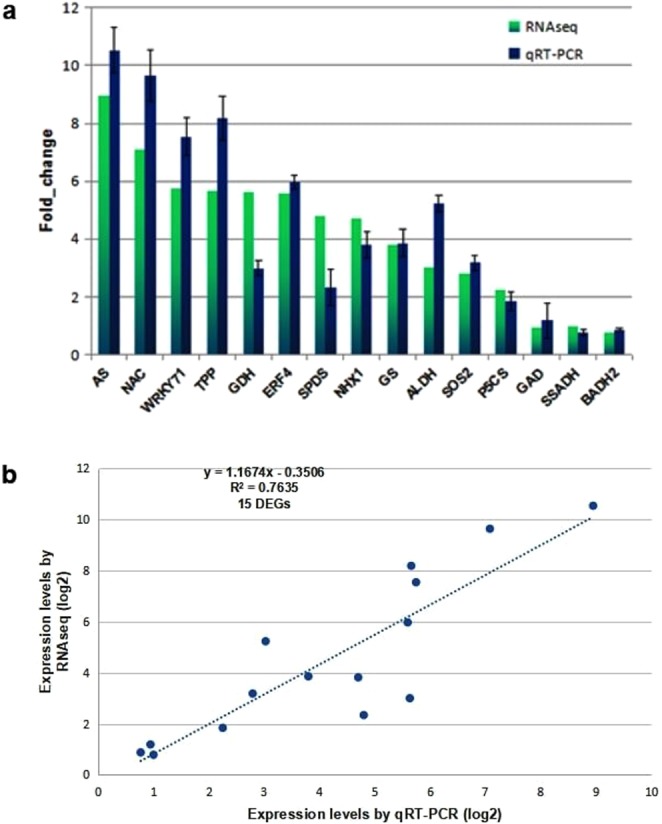
(**a**) qRT-PCR of selective up-regulated genes under 1 M salt treatment in *P*. *odorifer* (Legend:- AS: Asparagine synthetase, NAC: NAC TF, WRKY71: WRKY TF 71, TPP: Trehalose phosphate phosphatase, GDH: Glutamate dehydrogenase, ERF4: Ethylene responsive factor 4, SPDS: Spermidine synthase, NHX1: Na +/H + antiporter 1, GS: Glutamine synthetase, ALDH: Aldehyde dehydrogenase, SOS2: Salt overly sensitive-2, P5CS: 1-pyrroline 5 carboxylate synthase, GAD: glutamate decarboxylase, SSADH: Succinic semialdehyde dehydrogenase, BADH2: Betaine aldehyde dehydrogenase 2). (**b**) Scatter plot depicting the correlation between the RNAseq and qRT-PCR data. The figure shows expression changes (log2 fold) measured by RNAseq and qRT-PCR analyses of the selected genes. A linear trend line is shown.

To validate the RNAseq results of up-regulated genes, 15 salt-induced genes were selected randomly for analysis of quantitative reverse transcriptase polymerase chain reaction (qRT-PCR). The analyses of expression trends of genes from the qRT-PCR and RNAsequencing were largely consistent (Fig. 4a). To measure the correlation between the RNAseq and qRT-PCR results, a scatter plot of log2 fold values was constructed where we observed positive correlation coefficient (Pearson coefficient R^2^ = 0.7635) (Fig. 4b). Thus, the results confirmed the accuracy of transcriptomic profiling data.

### Enzyme assays for validation of up-regulated genes at protein levels

**Figure 5:**
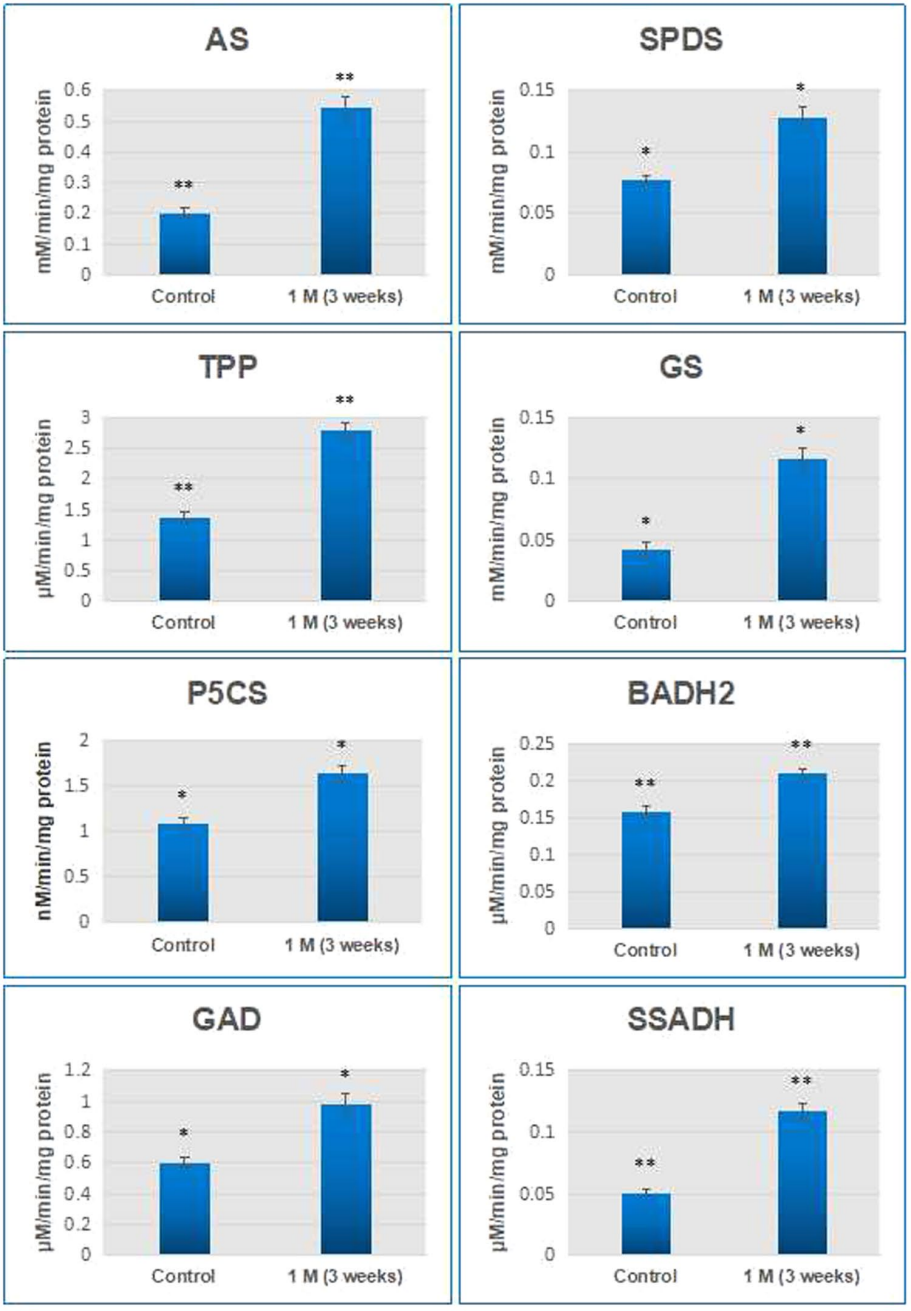
Enzyme activity assays of selected enzymes in *P*. *odorifer* under control and 1 M salt treatment. (* and ** on the histogram bar indicates significant changes in activity at p ≤ 0.05 and p ≤ 0.01 respectively).

To correlate the transcription activity with protein activity, enzyme assays were carried out in selected genes like *AS*, *TPP*, *SPDS*, *GS*, *P5CS*, *GAD*, *BADH2* and *SSADH* (Fig. 5). It was found that all the enzyme activities were up-regulated under salt treatment (e.g. AS activity was 2.74 fold, TPP was 2.04 fold, GS was 2.73 fold etc.). Nevertheless, as it can be observed from the data, there were differences in the magnitude of mRNA abundance and their corresponding enzyme activities. This variation is most probable because several factors regulate the gene expression levels in a cell such as transcription initiation rate, mRNA stability, translational efficiency and protein stability etc.^31^. Several researchers have also reported experimental evidences for the disparity between transcriptome and proteome data^32,33^. Thus, the results of enzyme activity largely correlate with RNAseq and qRT-PCR data.

### LC-MS based metabolomic profiling in *P*. *odorifer* under salt stress

For metabolite profiling analysis, we used the ProMetab software as it assumes peak detection, retention time correction and peak grouping^34,35^ for the assignment of mass peak to compound. ProMetab helps in selection of biologically significant biochemical reactions databases^36^ with Dirichlet-categorical prior distribution. Moreover, connection of observed mass peaks where particular metabolites are substrate or products of some known biochemical reactions is also facilitated during annotation for biologically meaningful interpretation of results^36^.

The analysis of individual metabolites synthesized during salt stress in *P*. *odorifer* was attempted through non-targeted metabolite profiling. It identified 8,159 compounds under both conditions (Supplementary Table S2, Sheet1). Out of these, identification of 3, 173 compounds was successful while 5,688 compounds were assigned as unknown since their identity could not be specified through the KEGG database. For all the identified compounds (3, 173), the average Bayesian probability was 0.677. Under salt treatment, considerable increase in amino acids, organic acids, sugars etc. was found above control. In terms of individual compounds, 2,3-dihydroxybenzoylserine (>84.0 fold) and Asn (>28.5 fold) recorded several highest increases followed by phenylethylamine (>20.16 fold), amifostine (>19.36 fold), styrene (>18.74 fold), mandelic acid (>17.35 fold), abscisic acid (ABA) (>6.79 fold), L-arginine (>6.04 fold), OAA (>2.84 fold), 1-pyrroline-5-carboxylate (P5C) (>2.82 fold) and tuliposide B (>2.3 fold) etc. At the same time, there was substantial decrease in the concentrations of several metabolites like Gln (0.398 fold), Glu (0.244 fold), alanine (0.609 fold), Asp (0.219 fold) and αKG (0.256 fold).

The metabolomic profiling showed increase in the levels of amino acids, organic acids, sugars etc. under salt treatment over control, which is often found in plants under stress. Noticeably, there was higher accumulation of two metabolites 2,3-dihydroxybenzoylserine and Asn. 2,3-dihydroxybenzoylserine is a breakdown product of enterobactin^37^ while Asn is an osmolyte synthesized via the activity of AS. Other metabolites were phenylethylamine, amifostine, tuliposide B, styrene, mandelic acid, ABA and others. Amifostine supposedly protects normal cells from DNA damage induction by inhibiting apoptosis and oxidative stress as reported in animals and green algae^38,39^. Tuliposide B, a plant metabolite, has antibacterial and antifungal properties^40^. Abundant presence of these compounds indicat that significant changes in the metabolic levels take place upon salt treatment in *P*. *odorifer*. They also reflect the tolerant nature of *P*. *odorifer*, which in turn may help them to survive and sustain the salt stress.

### Principal component analysis of metabolites in *P*. *odorifer*

Large datasets are summarized via PCA method to interpret principal component (PC) scores easily. This accounts for as much variance in the data as possible using the smallest number of PCs^41^. 2-D and 3-D score plots provide meaningful understanding of metabolomics data. For the interpretation of pattern of score plots, PCA biplots are used, that describe the metabolites contributing variance between samples. When general spectral trends (PCA) and group predictive spectral features (OPLS-DA) are jointly applied to spectral datasets^42^, they provide with valuable insights.

**Figure 6:**
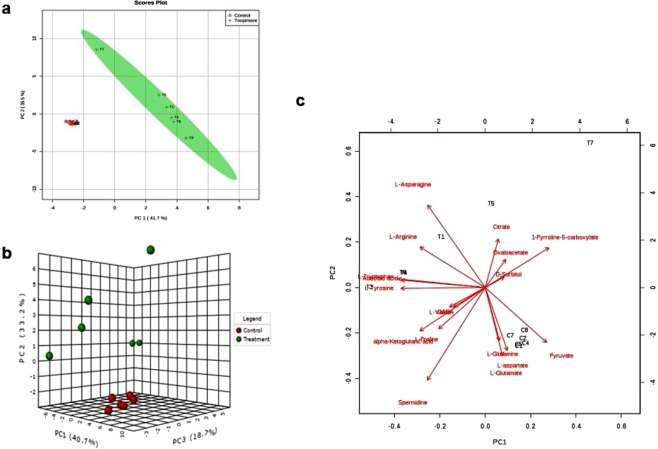
(**a**) 2-D Scores plot of leaf metabolite profiles of control and salt treated seedlings in *P*. *odorifer*. Each data point represents the metabolite profile of a single sample. The principal components 1 and 2 account for 76.9% variance in the data. (**b**) OPLS-DA 3-D score plot analysis of leaf metabolite profiles showing visible differences in metabolites under control and salt treatment in *P*. *odorifer*. (**c**) PCA biplot between the selected PCs under salt treatment in *P*. *odorifer*

Multivariate analysis of the metabolic response was performed to select the features that are differentially expressed under salt stress in *P*. *odorifer*. Figure 6a illustrates 2-D PCA scores of the two groups (control and treatment) by showing clear differentiation from each other. The principal components 1 and 2 are accounted for 76.9% of the variance in the data. Figure 6b shows orthogonal partial least squares discriminant analysis (OPLS-DA) 3-D score plot, representing visible differences in metabolites under control and salt treatment in *P*. *odorifer*. The PCA on metabolite data helped in the construction of a biplot (Fig. 6c) for the selected metabolites (Supplementary Table S3). The PCA biplot showed that several compounds including Asn, citrate, P5C, OAA, spermidine (Spd), Gln, Asp and others greatly contributed to the separation of controlled and treated samples. Among these selected metabolites, 13 were considered for MS/MS analysis where identities of 8 metabolites were successfully confirmed (Supplementary Table S4) (Supplementary Fig. S8a–g).

### Salt stress promoted accumulation of endogenous asparagine

The analysis of metabolites in the salt treated and control tissues revealed that the pool for Asn was the second highest amongst all the major metabolites found in plant tissues. Upon salt treatment, the pool for Asn increased to 28.5 fold compared to control and it accounted for 4.3% of the total amino acid pool. The pools of other metabolites involved in Asn pathway like arginine, OAA, P5C and αKG also increased (Supplementary Table S2, Sheet1) (Fig. 3). Salt treatment also led to minor increase in the pools of GABA and proline. Surprisingly, the pools for alanine, Gln, Glu, Asp and pyruvate decreased despite the genes involved in their synthesis (alanine synthesizing transaminase, GS, GOGAT, PK and aspartate kinase) were up-regulated (Supplementary Table S2, Sheet2). Therefore, it can be predicted that under salt stress conditions in *P*. *odorifer* these metabolites are synthesized and used to channelize the major metabolic pool towards the synthesis of Asn. Also, it is probable that insignificant increase in GABA (1.16 fold) and proline (1.15 fold) contents under salt stress could be because of the antagonistic effect of Asn. Similar results of enhanced Asn pool were reported in severe salt or drought stressed barley seedlings, soybean, *Coleus blumei*^43–45^, alfalfa^15^, pearl millet^46^ and wheat^47^. Several researchers have notified that Asn acts likewise to proline in plants under stressful conditions. Asn has been found accumulating to extents even greater than proline and hence serve as an additional marker^48^.

Various prospects have been stated to justify the mechanistic basis of Asn accumulation from a biochemical perspective particularly under stress. Asn is proposed to serve as carbon and nitrogen source for plants under stress specifically during recovery/rehydration^49^. Selective studies have suggested that Asn acts as ammonia detoxification agent and is produced when abiotic stresses lead to ammonia accumulation in plants. For instance, in *Arabidopsis*, increase in cellular free NH_4_^+^ and ASN2 mRNA levels, in a coordinated manner, can be observed during NaCl and cold stress^50^. In addition, Asn is also known as a key signaling molecule for maintaining the flow of N among the organs, thereby serving the role of a compatible osmolyte and hence controlling osmotic pressure of tissues^48^. It has also a proposed role in the photorespiratory N-metabolism in the leaves, through transamination reactions^29,50^. An equivalent role for most other metabolites in *P*. *odorifer* seems unlikely because their proportional concentrations under salt stress are not found significant enough. Therefore, based on the results of this study, it is interesting to suggest that Asn functions as compatible solute in cytosol of salt stressed *P*. *odorifer*, and contributes significantly to the osmotic adjustments of the cellular system.

### Combined evaluation of AS transcripts and Asn metabolite

Gln dependent AS is essentially a cytoplasmic enzyme, which catalyzes the synthesis of Asn from Gln and Asp via amide transfer. Under salt stress conditions in *P*. *odorifer*, we found heavily up-regulated *AS* (8.9 fold) compared to control and validated it by qRT-PCR. We found close relationship between the abundance of *AS* transcripts and free Asn levels in *P*. *odorifer* under salt stress, which has also been observed in *Arabidopsis thaliana*^51^ and sunflower^52^. This indicates that transcription is a relevant step in the regulation of Asn synthesis. Gilbert *et al*. (1998)^44^ state that under stressful conditions, accumulation of Asn is partially due to its *de novo* synthesis. This could be a consequence of induction of *AS* or AS enzyme activities. Our experiment showed that salt stress in *P*. *odorifer* dramatically induces *AS* expression and suggested that the important regulatory role is played at the transcription levels. The up-regulation of *AS* genes by salt and other abiotic stresses were also reported in maize^53^ and *Arabidopsis*^50^. In the present study, it was observed through combined results of transcriptome and metabolome analyses that the overall status of transcripts and respective metabolites related to the metabolic pathway of Asn in *P*. *odorifer* under salt stress justifies the correlation among themselves.

ABA is a widely known plant hormone that mediates plant responses to environmental stresses^54^. Likewise, most of the stress-inducible genes are induced when ABA is applied exogenously. In our experiment, we found that ABA level in *P*. *odorifer* under salt stress was nearly 6.8 fold higher than that in control tissues (Supplementary Table S2, Sheet1). This suggests that *AS* under salt stress in *P*. *odorifer* might be induced through ABA-dependent pathways as reported in the case of wheat *AS*^55^.

### AS enzyme

It was noticed that the level of AS enzyme activity under salt stress, was ~2.7 fold higher compared to those for control plants. AS is the primary enzyme responsible for the synthesis of Asn in plants^56^, but it is ill-characterized at biochemical levels in plants. The primary reason for this may be the high instability (short half-life) of asparagine synthetase and rapid turnover of Asn by AG thereby making it difficult to characterize it *in vitro*^57^. Other issues associated with biochemical studies of AS are the presence of endogenous natural inhibitors^58^, relatively high cytoplasmic AGs^59^ and GS activities^56^ competing for similar substrates.

### Na^+^ accumulation in *P*. *odorifer* in response to NaCl stress

**Figure 7:**
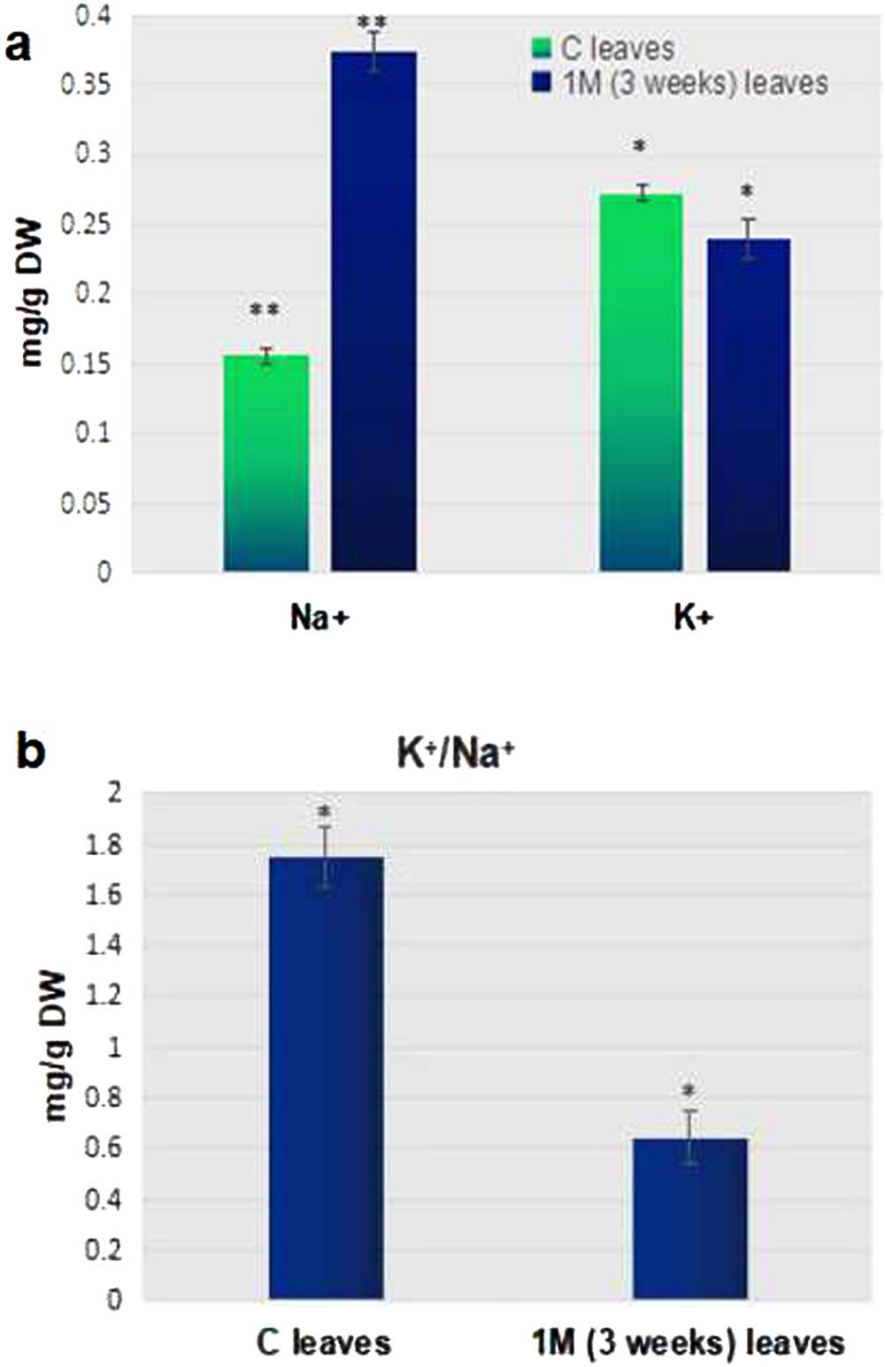
(**a**) Na^+^ and K^+^ levels in control ‘C’ and ‘treated’ (1 M (3 weeks)) leaf tissues of *P*. *odorifer*. (*and ** on a column indicates significant difference at p ≤ 0.05 and p ≤ 0.01 respectively). (**b**) K^+^/Na^+^ ratio in control and treated leaf tissues of *P*. *odorifer*. (*and ** on a column indicates significant difference at p ≤ 0.05 and p ≤ 0.01 respectively).

The estimation of Na^+^ and K^+^ contents in leaf tissues of control and 1 M salt treated seedlings showed that Na^+^ levels were significantly increased under salt treatment than control, whereas the concentration of K^+^ decreased. The Na^+^ contents in 1 M treated seedlings was 2.4 fold higher than the control, suggesting high accumulation of Na^+^ in leaf tissues of *P*. *odorifer* under salinity (Fig. 7a and b). Ion homeostasis via compartmentalization and osmoregulation are the major modes of survival for halophytic plants^60^. Accumulation of inorganic ions such as Na^+^ and Cl^−^ and osmolytes helps in osmotic adjustment in tolerant species^61^. These osmolytic compounds seldom interfere with normal metabolic reactions of plants since they are non-toxic even at higher cellular concentrations^62^. Similar trends could be seen in halophytic plants from Amranthaceae and Chenopodiaceae families^4,63^. In this study, it was found that Na^+^ levels were significantly increased than control under salt stress, whereas the concentration of K^+^ decreased under salt stress. It leads to lower K^+^/Na^+^ ratio (Fig. 7a and b). This indicates that *P*. *odorifer* seems to be selective for Na^+^ over K^+^. A lower K^+^/Na^+^ ratio under salt stress signifies lower selectivity for K^+^^64^, which has also been found in wild salt tolerant species of tomato and some halophytic plants^65^.

This observation was supported by RNAseq analysis. It revealed an up-regulated Na^+^/H^+^ antiporter (NHX1, 4.7 fold) along with V type H^+^ transporting ATPases (V-ATPase, 1.4 fold) which are mainly involved in compartmentalization of Na^+^ into vacuoles. A coordinated role of vacuolar Na^+^/H^+^ antiporters and V-ATPase helps sequestering Na^+^ in vacuoles in response to salt stress in plants^66^. A down-regulated HKT1 gene (0.793 fold) justifies the lower K^+^ levels under salt stress as seen in *Suaeda* species^5^. Selective sequestration of Na^+^ and Cl^−^ into vacuoles prevents the inhibitory effects on metabolic processes: a common phenomenon in tolerant plants^60,67^.

## Conclusions

In this study, we performed transcriptomics, metabolomics and enzyme activity studies in a halophyte *Pandanus odorifer* in order to explore the mechanism of tolerance to very high level of salinity. The integrative omics analysis revealed that both *AS*, and the metabolite Asn that are encoded by it, were highly up-regulated under salt stress. Similarly, the activities of the enzymes related to Asn synthesis were also high. Therefore, it could be established to large extent that Asn plays major role as an osmolyte and helps *P*. *odorifer* to manage the salt stress. Several other genes encoding TFs, ion channels, signaling molecules, ZFPs etc. were also highly up-regulated under salt stress. *AS* from *P*. *odorifer* could be considered as one of the genes in conferring salt tolerance in salt susceptible species. Further, the transcriptome repository that we have generated, will serve as a platform to enrich understanding about the molecular mechanism of primary and secondary metabolic pathways for salt stress tolerance in crops.

## Materials and Methods

### Plant material and salt stress treatment

About three month old healthy seedlings of *P*. *odorifer* at 4-leaf stage were collected and brought from coastal site Diveagar (Raigad, Maharashtra, India; Coordinates: 18°10′24″N 72°59′30″E) to the experimental site (Botany Department, Savitribai Phule Pune University, Pune, Maharashtra, India; Coordinates: 18°33′8″N 73°49′29″E) in pots. Seven of out of the collected plants were sown in each earthen pot of 18 cm diameter. With daily watering of 100 mM NaCl water, they grew for 1 month for acclimation. Various NaCl concentrations (Control [0 mM], 250 mM, 500 mM, 750 mM, 1 M, 1.5 M, 2.0 M, 2.5 M and 3.0 M) and time-points (0 day, 2 days, 1 week, 2 weeks, 3 weeks or wilting) were chosen for the experiment. After acclimation, salt treatment was initiated by an increment of 100 mM NaCl water daily till the final concentration was achieved. Plants were irrigated daily with 250 ml of salted water of selected concentration and control plants were given fresh water without any salt. Fresh water was flushed once a week to avoid salt accumulation at the roots. The experiment was arranged in randomized block design. The treatment was continued for three weeks or wilting or until the relative water content in leaves dropped by 50% of the control. The control and treated seedlings were gently uprooted, cleaned and the shoots and roots were separated during harvesting. Thereafter they were snap frozen in liquid nitrogen and stored at −80 °C until further analysis.

### Transcriptome analysis

#### RNA extraction and quality determination

Following the manufacturer’s protocol, total RNA was extracted from the leaf tissues of control and 1 M (3 weeks) salt-treated plants using Qiagen RNeasy Plant mini kit (Qiagen, Germany). The RNA samples were digested using DNase I at 37 °C for 30 min to remove potential genomic DNA contamination. The concentration and quality of each sample was determined using Nanodrop^®^ ND-1000 spectrophotometer (Thermo Fisher Scientific, USA) and running samples on 1% agarose gel. Furthermore, the quality of RNA was analyzed using Bioanalyzer 2100 (Agilent technologies, Singapore), and only the high-quality RNA samples with RNA Integrity number (RIN) more than 7 were selected. Equal quantities of RNA from three replicates were pooled for cDNA preparation and RNAseq analysis.

#### Sequencing and de novo assembly

The paired-end libraries were sequenced using Illumina HiSeq. 2500 system (Illumina, USA) by SciGenom, Kochi (India). The raw sequencing data were transformed by base calling into raw reads. The raw reads were cleaned using NGSQC Toolkit (v2.3.3 NIPGR, India)^68^ by discarding adapter sequences. Reads with unknown nucleotides more than 5% and all low quality (Q < 30) bases were filtered. These clean, high-quality reads were the basis for further analysis. The *de novo* transcriptome assembly of clean reads was performed using the short-read assembling program Trinity (v2.2)^69^ with default settings. The assembled contigs, longer than 200 bp, were clustered using CD-HIT tool (v4.6.1) to obtain non-redundant contigs^18^. Using BOWTIE2 (1.0.0)^70^ with default parameters, the transcript assembly was validated by back mapping high quality reads with assembled contigs. The validated assembly was used for normalization of mapped reads, and TPM (tags per million) and FPKM (fragments per kilobase per million) were obtained using RSEM software^71^. The edgeR Bioconductor package (v 3.5)^72^ was used for statistical analyses of expression of differential genes.

#### Functional annotation

Functional annotation of transcripts was performed using Blast2GO Pro^73^ software. Putative function was assigned to each transcript by using BLASTx-fast homology search against non-redundant (NR) Viridiplantae, [taxa:33090] from Jan 30, 2017 protein database, at the criteria of e-value < 1e^−10^. NR BLAST hits were used to derive associated Gene Ontology (GO) terms from UniProt database. The gene ontology (GO) terms and IDs of the functionally annotated unigenes were assigned on the basis of BLAST (e-value threshold of 1e^−06^) against plant GOslim set (http://agbase.msstate.edu/cgi-bin/tools/goslimviewer_select.pl). With nr annotation, Trianotate software (https://trinotate.github.io) was also used to obtain annotation of the transcripts with GO terms.

#### Differential gene expression analysis

The differentially expressed genes (DEGs) among control and salt treated samples were defined as those with an FDR (false discovery rate) ≤0.001 and log2 fold change (LFC) ratio ≥ 1 (FPKMs [fragments per kilobase per million] >2). Here, those transcripts which were absent and having FPKM values less than 10, from either sample were excluded from the study. The linkage hierarchical cluster analysis was performed on top 50 differentially expressed genes using Multiple Experiment Viewer (MEV v4.8.1). Using the log-transformed and normalized values of genes, heat maps were generated based on Pearson uncentered correlation distance.

### Validation of differential gene expression by qRT-PCR

The transcripts showing salt-induced expression and potential roles in the salt stress response were chosen for validation using qRT-PCR. Fifteen transcripts were selected with FPKM values > 5.5 in the salt treated samples. The expression analysis was performed for genes belonging to TFs, ion channels, Asn synthesis and osmolytes namely NAC, WRKY71, NHX1, SOS2, ERF4, AS, GS, GDH, trehalose phosphate phosphatase (TPP), P5CS, GOGAT, aldehyde dehydrogenase and spermidine synthase. Three independent biological replicates of each sample, along with three technical replicates each, were used in the analysis. Reverse transcription reactions were performed using Super Script III Reverse Transcriptase (Invitrogen, USA) with 2 μg total RNA according to the manufacturer’s instructions. The 5.8 s rRNA gene of *P*. *odorifer* was used as an internal control for all of the experiments. The primers for qRT-PCR (Supplementary Table S5) were designed using Primer3 software to amplify 100 to 150 bp regions of the chosen genes. qRT-PCR was performed using the Realplex real time PCR system (Eppendorf, USA) and SYBRGreen Mastermix (Biorad, USA). The 2^−ΔΔCt^ method^74^ was used to calculate, from cycle threshold values, the relative expression levels of the selected transcripts, normalized to the expression level of 5.8 s rRNA.

### Enzyme assays for validation at proteins level

Eight genes encoding the enzymes for respective metabolites, namely *AS*, *TPP*, *SPDS*, *GS*, *P5CS*, *GAD*, *BADH2* and *SSADH* were selected. Their enzyme activities were analyzed in control and 1 M (3 weeks) treated tissues. The experiments were carried out at 4 °C using 1 g of frozen (−80 °C) leaf tissues.

AS enzyme was extracted by following the methods of Romagni and Dayan^75^ and assayed for AS activity in accordance to ninhydrin colorimetric procedure by Sheng *et al*.^76^. Blank was prepared in a manner similar to test samples without enzyme. The standard samples containing Asn of different concentrations were measured at 340 nm for standard curve preparation.

TPP assay was performed by following the protocol of Umesh and Ramesh^77^. In this case, the enzyme activity was measured by determining the release of inorganic phosphate from the substrate trehalose-6-phosphate. The absorbance was read at 820 nm with the help of UV spectrophotometer (UV 1800, Shimadzu, Japan) and later was compared to a standard solution of inorganic phosphate. One unit of TPP activity is defined as the amount of enzyme that releases 1 nmol / min inorganic phosphate from trehalose phosphate at pH 7.0. Specific activity of TPP was expressed as nmol/mg protein/min.

Spermidine synthase (SPDS) enzyme was extracted using the methods of Sindhu and Cohen^78^. One unit of enzyme is defined as the amount of enzyme that forms 1 nmol of Spd/min under standard conditions. Standard curve was prepared and various concentrations of Spd used for it under identical conditions.

Previously mentioned method was followed for the extraction and carrying out the assay of the GS enzyme^79^. The enzymatic assay was performed by monitoring γ-glutamyl hydroxamate synthesis in the presence of NH_2_OH. Thereby γ-glutamyl hydroxamate formed which was measured at 540 nm using a spectrophotometer (UV 1800, Shimadzu, Japan). The comparison was initiated with standard γ-glutamyl hydroxamate in the presence of all components except ATP.

The protocol proposed by Kaikavoosi *et al*.^80^ was used to perform. The P5CS assay. Optical density of the assay mixture was measured at 340 nm light intensity for every 30 seconds for 3 min using the spectrophotometer (UV 1800, Shimadzu, Japan) against a blank without ATP.

Glutamate decarboxylase (GAD) activity measurement was conducted by referring to Renault *et al*.^81^. GAD activity was determined by quantifying the amount of GABA produced using GABase (Sigma, USA). The increase in OD at 340 nm was recorded using spectrophotometer (UV 1800, Shimadzu, Japan). External calibration curve method was used to determine the GABA concentrations. Bovine serum albumin was used as standard, stated by Bradford (976)^82^, to compute the total protein in the enzyme extracts.

Betaine aldehyde dhydrogenase 2 (BADH2) activity was measured following Weretilnyk and Hanson^83^ with some modifications. Proteins were estimated by the Bradford method^82^. The enzyme extract (~100 μg /reaction) was used for computing enzyme activity with GABald as substrate.

Succinic semialdehyde dehydrogenase (SSADH) from *P*. *odorifer* was extracted and purified by the method of Satya Narayan and Nair^84^. SSADH activity was determined according to Kumar and Punekar^85^. The reaction was initiated by addition of SSA (100 µM final concentration) and enzyme activity was continuously monitored at 340 nm and 25 °C in a spectrophotometer (UV 1800, Shimadzu, Japan). One unit of enzyme activity was defined as the amount of enzyme, which catalyzes the formation of 1 nmol NADH/min at 25 °C.

### Global metabolite profiling

Before running the samples, the liquid chromatography–mass spectrometry (LC–MS) system was evaluated several times for stability and reproducibility. Stability of the system was confirmed by Retention times (RT), mass accuracies and peak areas of the two selected extracted ion chromatograms (EICs) in the quality control (QC) samples. In all the QC runs, intensity coefficient variation of leucine (Leu) and isoleucine (Ile) was 0.148 and 0.145, respectively. Alignment of the EIC of Leu and Ile revealed least deviation in RT shift. There was 1 ppm of mass accuracy during data acquisition (Supplementary Fig. S9). These results demonstrated that the reliability and stability of the system were qualified for running the samples and data acquisition.

Non-targeted metabolic profiling was performed in control and salt-treated tissues of *P*. *odorifer*. The same leaf tissues used for RNAseq were used. The method of Kumar *et al*. (2015) was used for the same^86^. LC-MS and Ultra High Performance Liquid Chromatography (UHPLC) were employed to identify and quantify the metabolites under salt stress and control conditions. Six biological replicates each of control and treated tissues were considered for the analysis in both positive and negative modes. Initially two-step extraction procedure was carried out to obtain all possible metabolites with good chromatogram and coverage of all the components in the sample. For this, the tissue was finely powdered using liquid nitrogen and 100 mg of frozen homogenate was transferred to 1.5 ml centrifuge tube. Metabolites, from this tissue sample, were extracted with 400 ul of 70% ice cold methanol and 0.1% formic acid. This was followed by sonication (S450H; 400 W/37 kHz, Elmasonic, Germany) for 20 min and centrifugation at 15,000 *g* for 30 min 4 °C. The supernatant was filtered with 0.2 μ amicon filter (Millipore, Germany) and stored at −80 °C until further use.

The Accela™ UHPLC system (Thermo Fisher Scientific, USA), coupled online through heated electrospray ionization source (HESI), with the QExactive Orbitrap mass spectrometer (Thermo Fisher Scientific, USA), was employed for non-targeted metabolomics profiling. The sample injection volume was 1.5 μl. The metabolites were profiled using a C18 Hypersil Gold column (1.9 μm, 2.1 mm × 150 mm, Thermo Fisher Scientific, USA). The temperature of column oven was set to 40 °C while the sample manager was at 4 °C. The eluents A (water containing 0.1% formic acid) and B (acetonitrile containing 0.1% formic acid) were employed in the electrospray ionization-positive (ESI+) mode and electrospray ionization-negative (ESI−) mode. The flow rate was adjusted at 350 μl/min with a linear gradient elution over 15 min. From the start to 0.3 min, eluent B was held at 2%, linearly increased to 30% till 2 min, to 45% during next 5 min, and then to 98% in 12 min. Subsequently, eluent B was returned to 2% in 13.4 min; it was held for additional 1.2 min before returning to the initial conditions. Sequence of the sample was random. In the ESI + mode, the MS spray voltage was 4.2 KV, whereas it was 3.8 KV in the ESI− mode. The capillary temperature was set at 320 °C with the sheath gas at 45 arbitrary units and the aux gas at 12 arbitrary units. The tube lens was set to 50 V and the mass scan range was set from 80 to 1000 m/z. The resolution of the Orbitrap was set to 70,000.

The MS/MS data was collected with collision energy between 35 and 40 eV. Mass Frontier^TM^ spectral interpretation software (Version 7.0, Thermo Scientific, USA) was used to predict structure based fragmentation pattern of the selected metabolites. Fragments from QC, standard and Mass Frontier^TM^ for individual metabolites were matched (Supplementary Table S4) with the help of R programming code (Supplementary File S6).

Extracted ion chromatograms (EICs) of Leucine (Leu) and Isoleucine (Ile) were selected in order to verify the resolution of the mass spectrometer, intensity deviation, ppm error and retention time shift. Moreover, to validate the stability of system, the retention times, mass accuracies and peak areas of these two selected EICs in the QC samples were also considered.

### Metabolome data analyses of *P*. *odorifer* under salt stress

ProteoWizard v3.0^87^ was used to extract raw metabolomics data into open interchangeable mzXML format. ProbMetab^34,35^ was employed for peak detection, retention time correction and peak grouping in order to perform mass peak to compound assignment. For the positive (ESI+) and negative (ESI−) ionization modes, the data were processed separately and combined later. The R-script, used for present purpose, is provided as Supplementary File S3. The entire procedure was divided into three steps:

Ion annotation extraction and database matching (pre-processing): Filtration of each ion redundant form, a complexity reduction step, was performed using CAMERA^35^ and mzMatch^88^. The Comb2 + algorithm^36^ was used to combine individual ion annotations. Based on biological knowledge, enhanced ion identification was carried out with the KEGG databases (http://www.genome.jp/kegg/). Genome scale metabolic reconstruction was performed via integration to metabolism repository MetExplore^89^. Comb2+ strategy was used for downstream processing.

Probability modelling and estimation: Candidate formulae, for a given mass, were filtered through relative isotopic abundance measurement. The likelihood of each mass to compound assignment using mass accuracy and isotopic pattern, when present, were calculated by weight^90,91^. The relation between compounds, given by the reaction present in the biological database, was codified by design.connection. Assignments of posterior probabilities were calculated using Gibbs sampler algorithm^92^.

Comprehensive output representation (post-processing): Calculations of correlations and partial correlations and cross reference with reactions were performed. Necessary objects were loaded to generate the table. Metabolomic data processing, normalization and multivariate statistical analysis were performed using the web based tool MetaboAnalyst 3.0 (http://www.metaboanalyst.ca)^93,94^. This tool also helped in generation of fold change (FC) plot, volcano plot and PCA analysis. Since column-wise normalization significantly changes the absolute values, FC was calculated as the ratio between two group means. It was done using data before applying column-wise normalization. For paired analysis, the MetaboAnalyst program counts the number of pairs with consistent change above the given FC threshold.

### Quantification of K^+^ and Na^+^ content of seedlings after treatment

Shoot samples were harvested from the seedlings, treated with 1 M NaCl for 3 weeks and control, and dried at 80 °C to constant weight in oven. Then the dried tissues were ground into fine powder. Tissue powders (0.1 g) were mixed with 10 mL HNO_3_: H_2_SO_4_ (3:1) and incubated at 150 °C for 6 h. Three biologically independent replicates were prepared. Thereafter, the Atomic Absorption Spectrophotometer (AA240; Agilent Technologies, USA) was used to measure K^+^ and Na^+^ concentrations.

### Statistical analysis

Average values and standard deviations were calculated in accordance with the experimental data. Error bars, in figures, represent standard error and each data point means ± SE of at least three experiments. One/two tailed Student’s t-test with unequal variance was used to test the significance of the data.

## Supplementary information

Supplementary information

Supplementary Table S1

Supplementary Table S2

## Data Availability

The raw data files generated during the present study have been deposited in the NCBI repository as *P*. *odorifer* RNA sequencing raw data files (SRA BioProject ID: PRJNA 389238 Sample ID: SRR5927127, SRR5927128) (Embargo period: 30 June 2020).
