# Supplementary material for: Integrative omics analysis in Pandanus odorifer (Forssk.) Kuntze reveals the role of Asparagine synthetase in salinity tolerance
Source: Sci Rep. 2019 Jan 30;9:932. doi: 10.1038/s41598-018-37039-y (PMC6353967; doi:10.1038/s41598-018-37039-y)
Supplement: Supplementary file 1 — Supplementary information [file 41598_2018_37039_MOESM1_ESM.pdf]

Supplementary information

Integrative omics analysis in *Pandanus odorifer* (Forssk.) Kuntze reveals the role of Asparagine synthetase in salinity tolerance

Deo Rashmi[1¥], Vitthal T. Barvkar[1¥ €], Altafhusain Nadaf[1*], Swapnil Mundhe[2] and Narendra Y. Kadoo[2]

1 Department of Botany, Savitribai Phule Pune University, Pune 411007, India
2 Biochemical Sciences Division, CSIR-National Chemical Laboratory, Pune 411008, India

* Corresponding author e-mail: abnadaf@unipune.ac.in
€ Co-corresponding author e-mail: bvitthal@unipune.ac.in

Supplementary Information contains: Supplementary Figure, Supplementary Tables and Supplementary File

Supplementary Figure legends:

Supplementary Figure S1: Assembled transcript length distribution of salt treated *P. odorifer*

Supplementary Figure S2: BLASTX sequence similarity distribution in salt treated *P. odorifer*.

Supplementary Figure S3: GC content distribution of assembled transcripts under control and 1 M salt treatment in *P. odorifer*.

Supplementary Figure S4: BLASTX e-Value distribution of hits generated using nr database in *P. odorifer*

Supplementary Figure S5: Top hit distribution of matched unigenes among different species generated using BLAST2GO.

Supplementary Figure S6: Gene ontology of DEGs under 1 M salt treatment in *P. odorifer*. a- Biological processes, b- Cellular components and c- Molecular functions related genes.

Supplementary Figure S7: Some of the GO terms for up-regulated transcripts in 1 M salt treated *P. odorifer*.

Supplementary Figure S8a: MS/MS spectra of Asn showing spectra of standard and pooled sample.

Supplementary Figure S8b: MS/MS spectra of ABA showing spectra of standard and pooled sample.

Supplementary Figure S8c: MS/MS spectra of Spd showing spectra of standard and pooled sample.

Supplementary Figure S8d: MS/MS spectra of αKG showing spectra of standard and pooled sample.

Supplementary Figure S8e: MS/MS spectra of GABA showing spectra of standard and pooled sample.

Supplementary Figure S8f: MS/MS spectra of Gln showing spectra of standard and pooled sample.

Supplementary Figure S8g: MS/MS spectra of Glu showing spectra of standard and pooled sample.

Supplementary Figure S9: Overlaid EIC of Leu and Ile from pooled QC samples of *P. odorifer*.

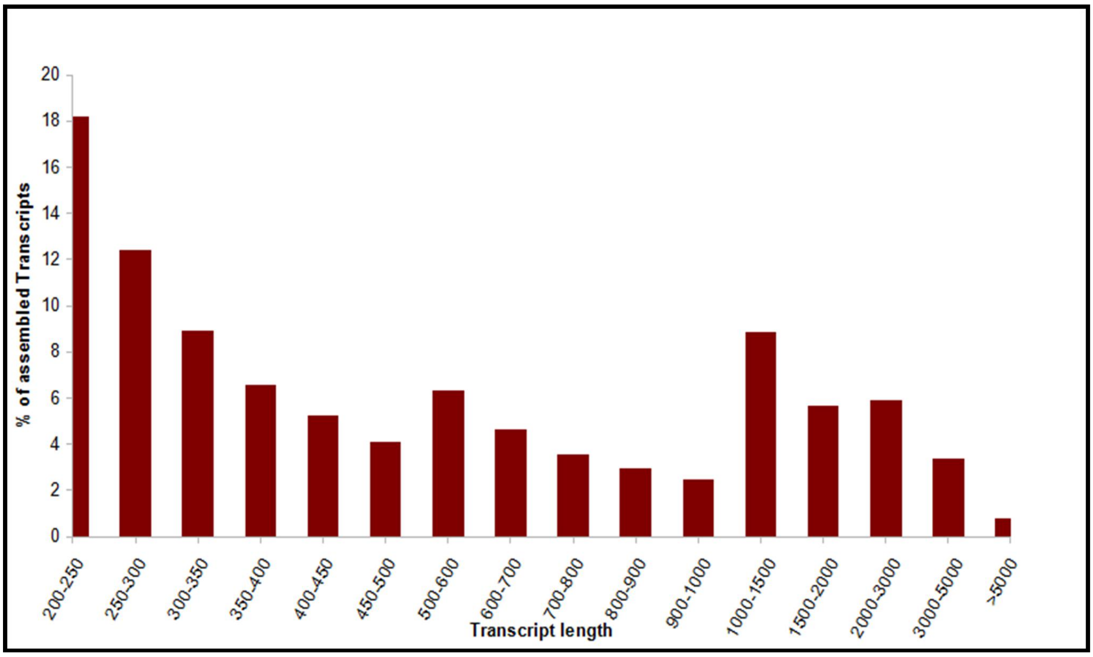

Supplementary Fig. S1: Assembled transcript length distribution of salt treated *P. odorifer*

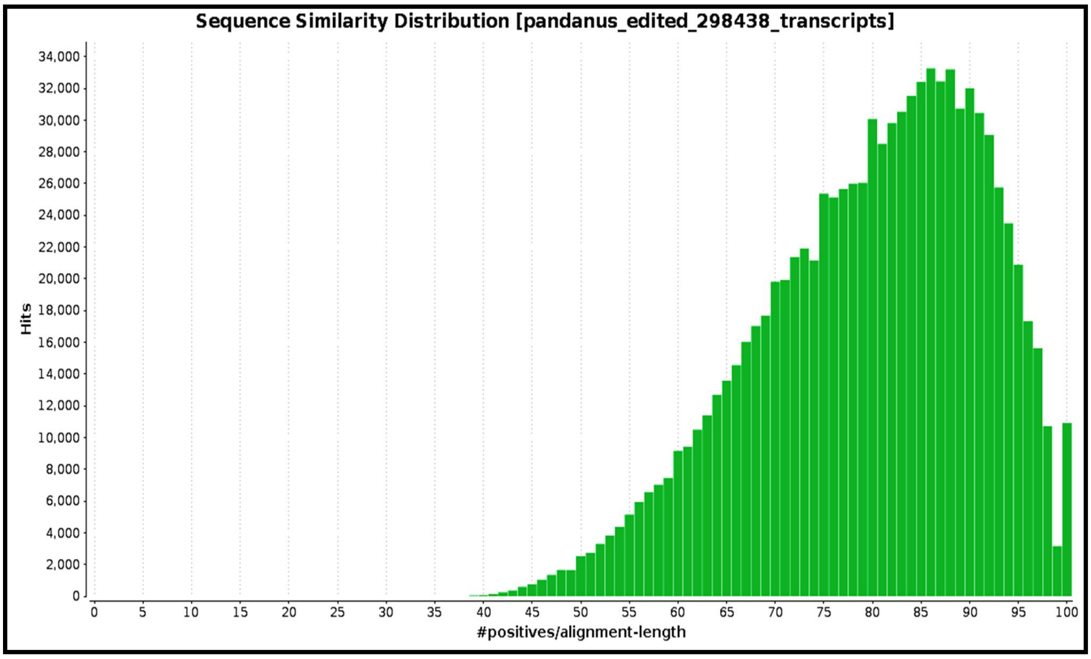

Supplementary Fig. S2: BLASTX Sequence similarity distribution in salt treated *P. odorifer.*

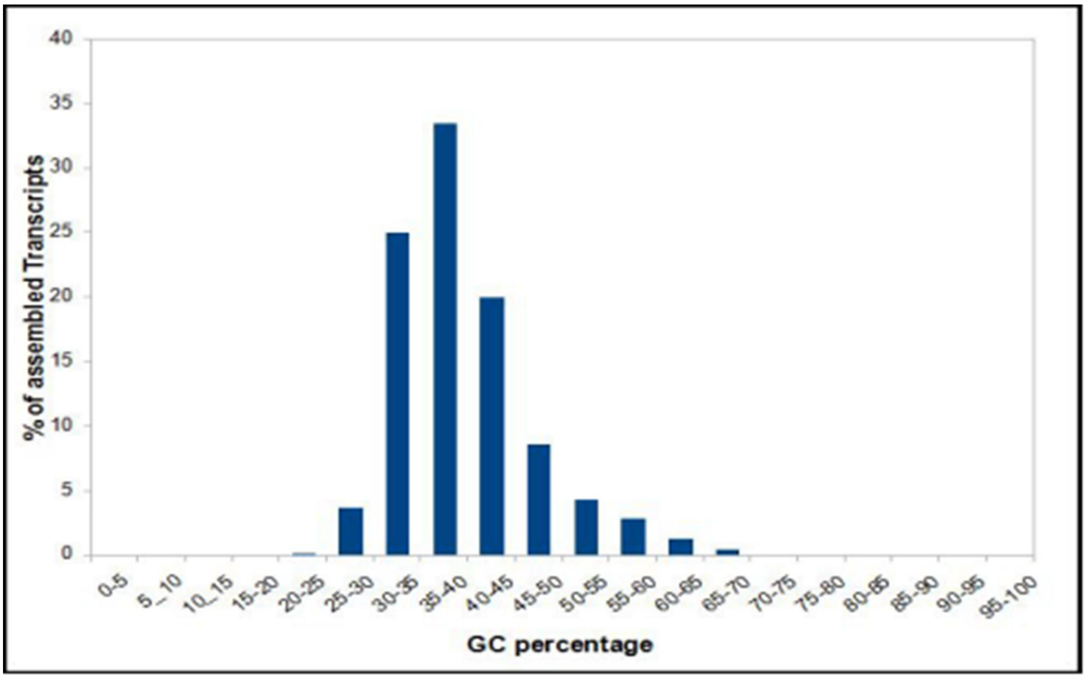

Supplementary Fig. S3: GC content distribution of assembled transcripts under control and 1 M salt treatment in *P. odorifer.*

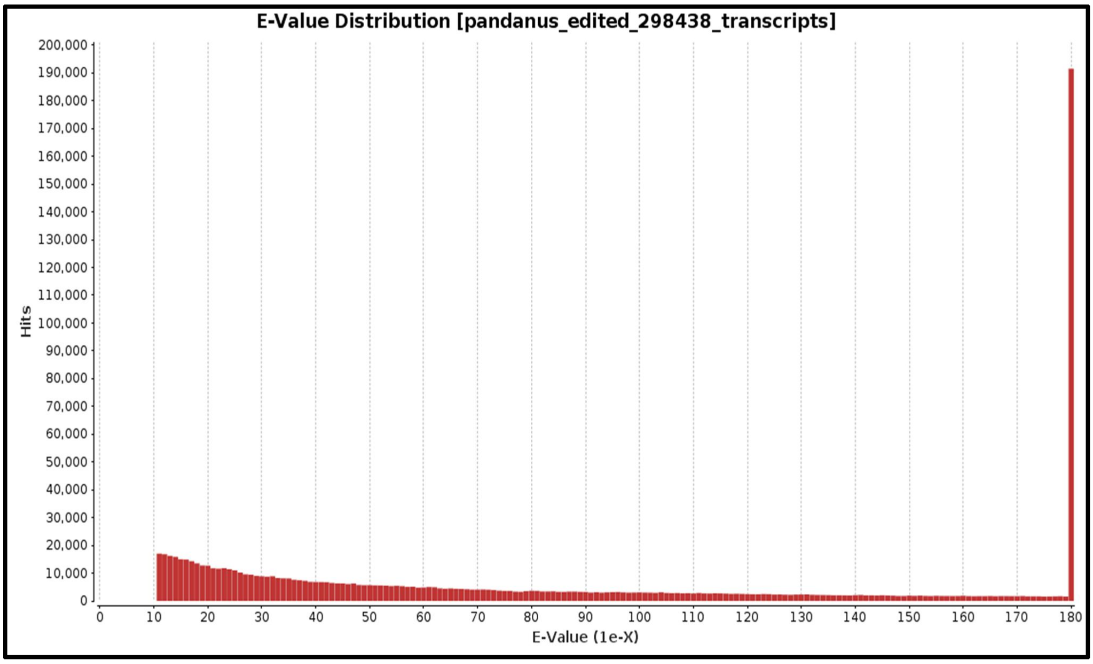

Supplementary Fig. S4: BLASTX E-Value distribution of hits generated using Nr database in *P. odorifer*

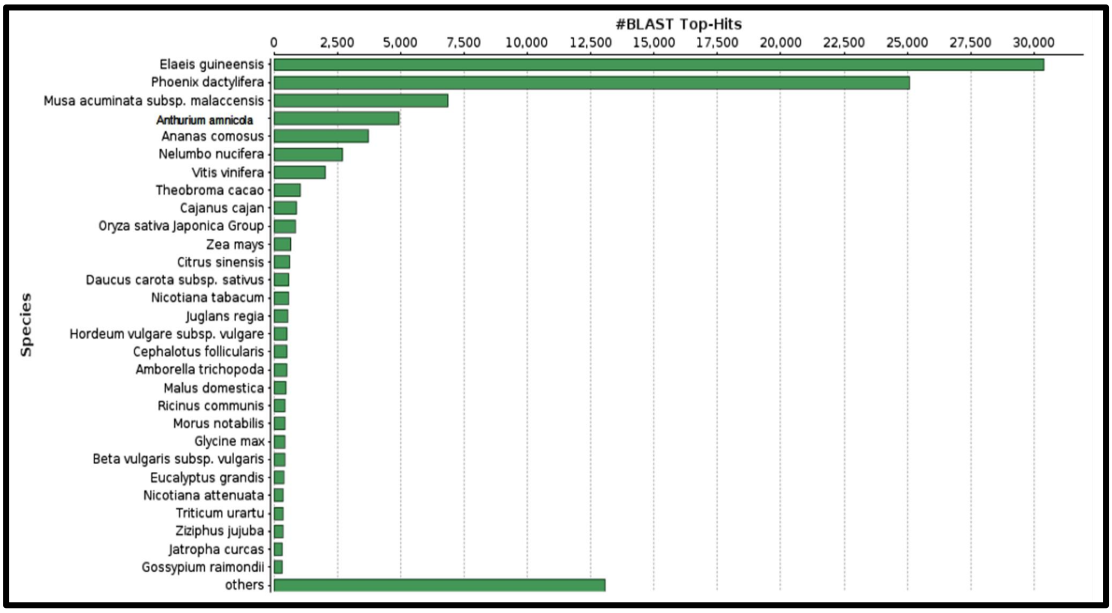

Supplementary Fig. S5: Top hit distribution of matched unigenes among different species generated using BLAST2GO.

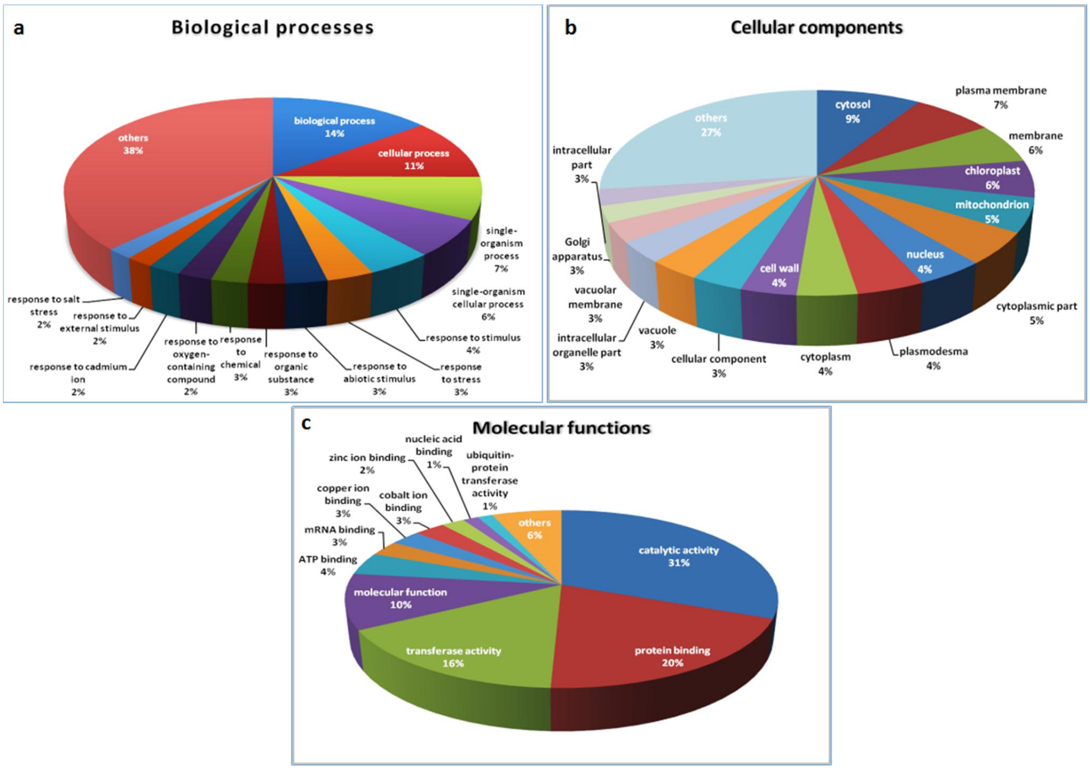

Supplementary Fig. S6: Gene ontology of DEGs under 1 M salt treatment in *P. odorifer.* a- Biological processes, b- Cellular components and c- Molecular functions related genes.

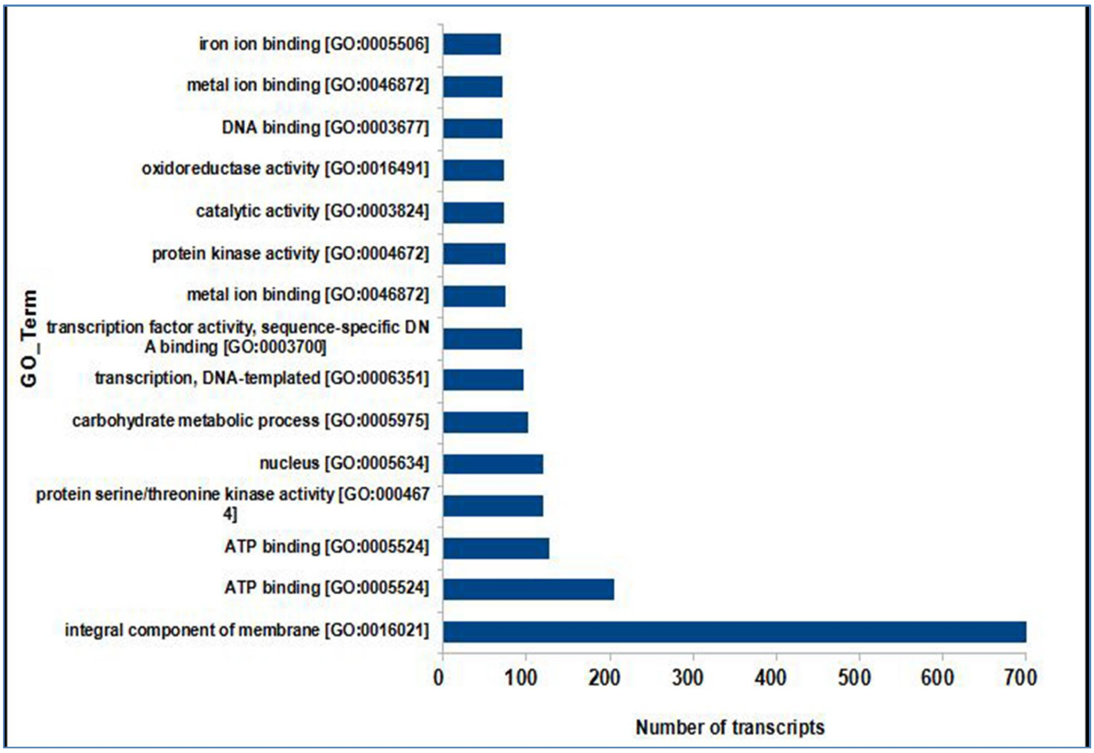

Supplementary Fig. S7: Some of the GO terms for up-regulated transcripts in 1 M salt treated *P. odorifer.*

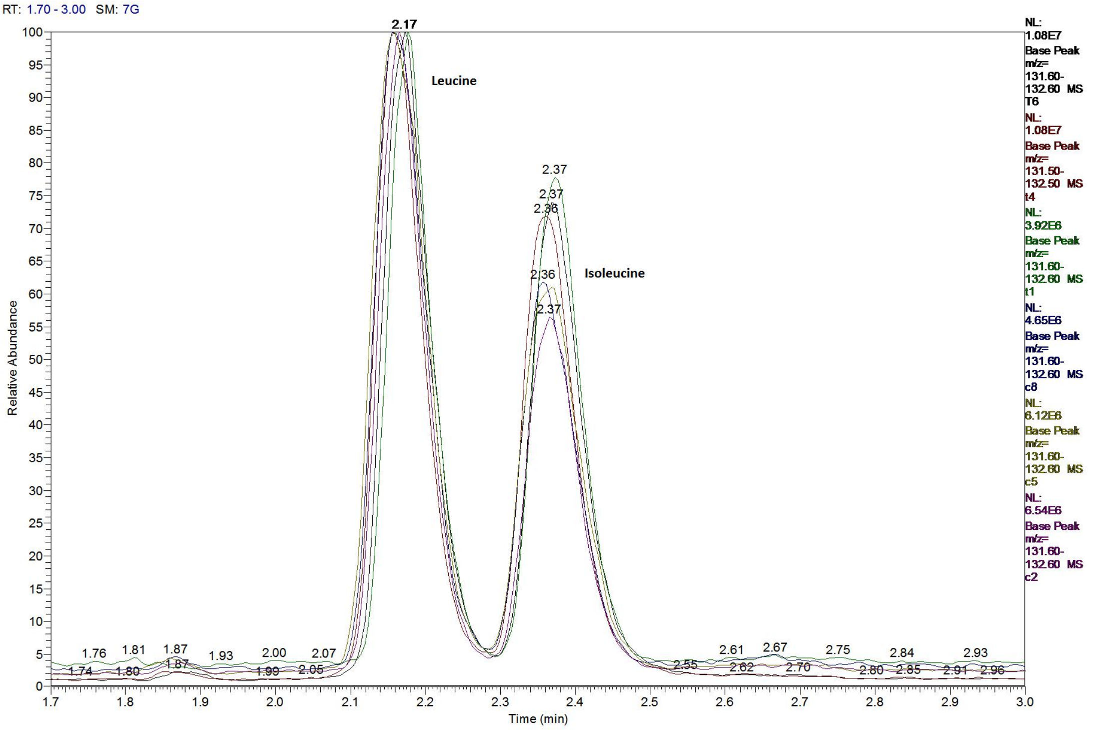

Supplementary Fig. S9: Overlaid EIC of Leu and Ile from pooled QC samples of *P. odorifer*

List of Supplementary tables

Supplementary Table	Sheet No.	Title
**Table S1**	1	Assembled IDs with sequence and length
**Table S1**	2	edgeR DE results for control and treatment
**Table S1**	3	KEGG pathway summary of analyzed transcript
**Table S2**	1	List of metabolites found under treatment and control upon metabolome analysis (values in C and T columns display area under peak for control and treatment, 6 replicates each).
**Table S2**	2	Pooled sample MS/MS
**Table S2**	3	Standards MS/MS
**Table S3**		Important features identified for PCA analysis in salt treated *P. odorifer*
**Table S4**		Selected molecules for MS/MS analysis
**Table S5**		List of primers used in qRT-PCR of up-regulated genes under salt stress in *P. odorifer*

Supplementary Table S1: Provided as separate excel document

Supplementary Table S2: Provided as separate excel document

Supplementary Table S3: Important features identified for PCA analysis in salt treated *P. odorifer*

Name	KEGG compound	Proposed mass	Average area under peak (Control)	Average area under peak (Treatment)	Fold change (FC)
L-Asparagine	C00152	132.0540900	844341.567	24081761.500	28.5210
L-Tryptophan	C00078	204.0906100	11824597.470	100697015.000	8.5159
Abscisic acid	C13546	250.1568415	398267.050	2705277.183	6.7926
Pyruvate	C00022	88.0152469	96624231.670	14398247.500	0.1490
L-Arginine	C05945	254.0768595	8136395.486	49159944.370	6.0420
L-Tyrosine	C00082	181.0746388	12539369.320	74726468.620	5.9593
L-aspartate	C00049	133.0375000	6400384.333	1406408.200	0.2197
L-Glutamate	C00025	147.0536553	75476502.330	18425118.330	0.2441
Citrate	C00158	192.0266132	6189475.630	19278360.050	3.1147
Oxaloacetate	C00036	132.0050338	1143858.167	3253677.000	2.8445
P5C	C03912	113.0478588	1329330.500	3758383.900	2.8273
L-Glutamine	C00064	146.0696756	42908013.830	17095242.330	0.3984
α-Ketoglutarate	C00026	146.0220697	7398395.667	12970709.870	1.7532
Spermidine	C00315	145.1578645	803219.500	1179153.837	1.4680

GABA	C00334	103.0635417	18605623.830	21597955.170	1.1608
L-Alanine	C00041	89.0479685	2199471.500	1340349.017	0.6094
L-Proline	C00148	115.0634633	42003018.720	48450827.090	1.1535
D-Sorbitol	C00794	182.0779525	2686748.201	4517532.696	1.6814
L-Isoleucine	C00407	131.0938118	89434.431	129808.687	1.7659
L-Valine	C00183	117.0790863	4829939.777	5583826.877	0.4363

Supplementary Table S4: Selected molecules for MS/MS analysis

**Kegg ID**	**Name**	**Formula**	**Proposed mass**	**Calculated mass**	**Adduct (M+1, M-1, M+23)**	**ppm error**	**No. of fragments match**	**MS/MS fragments**
C00152	L-Asparagine	$C_4H_8N_2O_3$	132.0541	133.0608	133.0607	0.638	6	70.0294, 74.0243, 87.0558, 88.0397, 116.0345, 133.0608
C06082	Abscisic acid	$C_{15}H_{20}O_4Na$	264.1362	287.1254	287.1252	0.5805	4	109.0651, 119.0859, 135.0805, 163.0754
C00026	α-Ketoglutaric acid	$C_5H_6O_5$	146.0221	145.0131	145.0131	0.0823	6	73.0281, 99.0072, 101.0231, 102.0266, 129.0181
C00315	Spermidine	$C_7H_{19}N_3$	145.1579	146.1652	146.165	1.237	11	72.0814, 73.0847, 75.0923, 84.0813, 84.0863, 112.1124, 113.0058, 129.1388, 130.1423, 146.1652, 147.1687
C00334	γ-Aminobutyric acid	$C_4H_9NO_2$	103.0635	102.055	102.0549	0.9769	8	59.0124, 740233, 74.9605, 101.0231, 102.0548, 103.0388, 103.0576, 103.919
C00041	L-Alanine	$C_3H_7NO_2$	89.04797	90.55	90.0554	4.4844	2	72.04, 90.05
C00064	L-Glutamine	$C_5H_{10}N_2O_3$	146.0697	145.0608	145.0608	0.0489	8	84.0442, 89.0231, 102.0548, 118.9651, 127.0501, 128.0341, 145.0607, 146.0448
C00025	L-Glutamic acid	$C_5H_9NO_4$	147.0537	146.0448	146.0448	0.2069	12	74.0235, 82.0287, 84.0443, 85.028,

Kegg ID	Name	Formula	Proposed mass	Calculated mass	Adduct (M+1, M-1, M+23)	ppm error	No. of fragmen ts match	MS/MS fragments
								101.0229, 102.0548, 103.0516, 103.0581, 127.0501, 128.0341, 145.0607,146 .0447
C03912	1-Pyrroline-5-c arboxylate	$C_5H_7NO_2$	113.0479	114.0557	-	-	-	-
C01044	N-Formyl-L-as partate	$C_5H_7NO_5$	161.0328	162.0407	-	-	-	-
C00022	Pyruvate	$C_3H_4O_3$	88.01523	87.00741	-	-	-	-
C00036	Oxaloacetic acid	$C_4H_4O_5$	132.005033 8	130.9972	-	-	-	-
C00062	L-Arginine	$C_6H_{14}N_4O_2$	174.110603 8	119.0498	-	-	-	-

Supplementary Table S5: List of primers used in qRT-PCR of up-regulated genes under salt stress in *P. odorifer*

Sr No.	Gene	Primer	Sequence	Amplification length (bp)	PCR efficiency (%)
1.	Asparagine syntheatse	AS_FP	CAGTTCAGTGATGGCGTTGG	152	103.7
		AS_RP	ACTGTTCCAGGAGGTCCAAGT		
2.	Glutamine synthetase	GS_FP	GGTATGGGATTGAGCAGGAA	166	96.0
		GS_RP	TCTCATTACAAGGCCTGCCT		
3.	Aldehyde dehydrogenase	ALDH_FP	ACCTTGTCAATGTCCATGTGG	157	100.0
		ALDH_RP	CTCAGCGGTCTTCATGACAA		
4.	Glutamate dehydrogenase	GDH_FP	CCATATGGATTCCTCCGATG	200	102.9
		GDH_RP	GCTTGTGGTCTTCTTGCTCC		
5.	Trehalose phosphate phosphatase	TPP_FP	CTACATGGAGGCAACCGAT	140	94.7
		TPP_RP	GAGAATGTGCTGGCCAATGA		
6.	P5C synthase	P5CS_FP	GGGATAATGACAGTTTGGCAG	200	100.8
		P5CS_RP	AAGAGGAGGAATGACTGCTAAA		
7.	Spermidine synthase	SPDS_FP	CCTGGTGGTGTTCTTTGTAAC	139	97.6
		SPDS_RP	TGTTCCTACGTACCCTAGTG		

8.	NAC TF	NAC_FP	TACTGGAAGGCAACAGGGG	130	98.4
		NAC_RP	CAAGACTGACTGGATCATGCA		
9.	Na^+-H^+ antiporter	NHX1_FP	TGATTGGGGGAAGCAAGTAG	152	97.0
		NHX1_RP	TTCATCCACCGGCTCTTCTC		
10.	Salt overly sensitive	SOS1_FP	TGCAAGTTTCTGCCATCAAGTG	147	97.9
		SOS1_RP	TGCTAAGCTGGCCAGAAAATTT		
11.	Glutamate decarboxylase	GAD_FP	GTGGGATCTTCAGAAGCAAT	170	101.21
		GAD_RP	CGTAATATCCCTCTGTCAACT		
12.	WRKY TF	WRKY_FP	GTTCCTGCAAACCCTACCG	104	93.6
		WRKY_RP	CATCCAAAACAACCAACCATGAT		
13.	ERF4 TF	ERF4_FP	GAGGCGGACGTTCTCGG	143	93.3
		ERF4_RP	CTTATGTGGATCGCGGATCT		
14.	BADH2	BADH_FP	TGCTTTGAGTACTTTGCAGA	91	98.33
		BADH_RP	GAAATACATCTCAGAGAAC		
15.	SSADH	SSA_FP	TTGGTGGTAAAAGGCACAG	113	100.02
		SSA_RP	GGACGAGAAGGATCGA		
16.	5.8s rRNA	5.8s_FP	ACTCTCGGCAACGGATATCTA	120	94.23
		5.8s_RP	CGCAACTTGCGTTCAAA		

Supplementary File F- R-Script used for Metabolite analysis

```
#
#These three library
library(ProbMetab)
library(xcms)
library(CAMERA)

#setting one folder inside which positive and negative subfolders should be there

xsetP <- xcmsSet("G:/Vitthal/Pandanus/pandanus_new_meta_analysis/SUBANA/POS/",
method='centWave', ppm=1, peakwidth=c(5,20),
prefilter=c(3,100),snthresh=3,integrate=1, mzdiff=0.00005,fitgauss=FALSE,
verbose.columns=TRUE)

xsetN <- xcmsSet("G:/Vitthal/Pandanus/pandanus_new_meta_analysis/SUBANA/NEG/",
method='centWave', ppm=1, peakwidth=c(5,20),
prefilter=c(3,100),snthresh=3,integrate=1, mzdiff=-0.00005,fitgauss=FALSE,
verbose.columns=TRUE)

# align retention times across samples, grouping and integration
```

```
xsetP1 <- retcor(xsetP, method='obiwarp', plottype="none", profStep=1) #positive mode
xsetPnofill <- group(xsetP1, bw=5,    mzwid=0.015)
xsetP2 <- fillPeaks(xsetPnofill)

xsetN1 <- retcor(xsetN, method='obiwarp', plottype="none", profStep=1) #negative mode
xsetNnofill <- group(xsetN1, bw=5,    mzwid=0.015)
xsetN2 <- fillPeaks(xsetNnofill)

#Camera step for positive and negative

an <- xsAnnotate(xsetP2)
an <- groupFWHM(an, perfwhm = 0.6)
an <- findIsotopes(an, mzabs = 0.01)
an <- groupCorr(an, cor_eic_th = 0.75)
anP <- findAdducts(an, polarity="positive")

an <- xsAnnotate(xsetN2)
an <- groupFWHM(an, perfwhm = 0.6)
an <- findIsotopes(an, mzabs = 0.01)
an <- groupCorr(an, cor_eic_th = 0.75)
anN <- findAdducts(an, polarity="negative")

#combining positive and negative mode

ionAnnotP <- get.annot(anP, allowMiss=TRUE, minint=1000)
ionAnnotN <- get.annot(anN,    polarity="negative", allowMiss=TRUE, minint=1000)
comb2plus <- combineMolIon(ionAnnotP, ionAnnotN)

#Databse search

DB <- KEGGcpds

reactionM <- create.reactionM(DB, molIon=comb2plus, ppm.tol=10)

wl <-weightM(reactionM, useIso=FALSE)
w <- design.connection(reactionM)

# Probability calculations
x <- 1:ncol(wl$wm)
y <- 1:nrow(wl$wm)

conn <- gibbs.samp(x, y, 5000, w, wl$wm)

system.time(ansConn <- export.class.table(conn, reactionM, comb2plus, html=FALSE))

write.csv(ansConn$classTable, file="result.csv")
```
